# *In vitro* analysis of probiotic characteristics of gut-associated bacteria from *Solea solea*

**DOI:** 10.3389/fvets.2025.1581675

**Published:** 2025-06-04

**Authors:** Nadia Hussain, Afreen Fatima Mirza, Fatima Muccee, Amal H. I. Al Haddad, Muhammad Imran Afzal

**Affiliations:** ^1^AAU Health and Biomedical Research Center, Al Ain University, Abu Dhabi Campus, Abu Dhabi, United Arab Emirates; ^2^Department of Pharmaceutical Sciences, College of Pharmacy, Al Ain University, Al Ain Campus, Al Ain, United Arab Emirates; ^3^School of Biochemistry and Biotechnology, University of the Punjab, Lahore, Pakistan; ^4^Chief Operations Office, Sheikh Shakhbout Medical City (SSMC), PureHealth, Abu Dhabi, United Arab Emirates

**Keywords:** aquaculture, bile salts, cell adhesion, hemolysis, blue agri-economy

## Abstract

**Introduction:**

One of the major challenges hindering blue agri-economy of Pakistan, is the extensive use of antibiotics and chemotherapeutics in aquaculture. A sustainable alternative is the supplementation of fish feed with non-pathogenic and non-invasive probiotics. In this study, bacteria associated with gastrointestinal tract (GIT) of fish *Solea solea* were isolated and characterized for probiotic potential.

**Methods:**

Bacterial isolation was conducted from the gut using serial dilution method and Mueller-Hinton Agar (MHA) medium. Isolates were characterized through biochemical analysis and 16S rRNA gene sequencing. Analysis of, intestinal cell adhesion efficiency, tolerance to bile salts, NaCl and pH, survivability in simulated gastric conditions, antibiotic sensitivity profiling, heat shock tolerance, antimicrobial activity of bacteria against *Staphylococcus aureus* and *Pseudomonas aeruginosa*, hemolytic activity, cholesterol assimilation potential and resistance against antibiotics. i.e., azithromycin, erythromycin, amoxil, ciprofloxacin and velosef, was performed.

**Results:**

Five isolates were identified as *Lacticaseibacillus rhamnosus, Enterococcus faecium, Bacillus amyloliquefaciens, Bacillus subtilis*, and *Bacillus cereus*. All bacteria were fast growing. Optimal growth was observed at pH 5. All isolates demonstrated growth in simulated gastric medium. They exhibited γ-hemolysis, survived heat shock treatment at 100°C, and showed good cholesterol degradation efficiency (ranging between 26.77 and 83.44 mg/dL). Optimum cell adhesion potential was recorded at 90 min. i.e., 119–129 CFUs. All isolates were sensitive to antibiotics with sensitivity order velosef > ciprofloxacin > amoxil > erythromycin and azithromycin.

**Conclusion:**

Due to these probiotic characteristics, current study bacteria might be good candidates for antibiotics replacement in aquaculture.

## 1 Introduction

According to the Pakistan Economic Survey 2023–2024, aquaculture contributed approximately 2.4% to the country's gross domestic product (GDP). Recognizing its economic potential, provincial governments have allocated significant resources, including 429 million Pakistani rupees (PKR) for projects in Gwadar, 9.7 billion PKR in Punjab, and 5 million PKR in Balochistan (1). The sector also plays a crucial role in foreign exchange earnings, with fish import revenue fluctuating between 296 million USD (2009–2010) and 394 million USD (2016–2017) (2). In response to rising population demands, aquaculture has emerged as an essential source of animal protein, employing approximately 390,000 individuals across Pakistan (3). The country currently has a total fish pond area of 60.47 thousand hectares and 13,000 fish farms (3).

Despite these contributions, Pakistan's aquaculture sector faces considerable challenges, including outdated and unhygienic technologies, poor management practices, and environmental concerns. These deficiencies have prompted intermittent export bans by the European Union (4). Additionally, the sector lags behind regional counterparts, with an annual growth rate of just 2.5% compared to India's 8% (5). Key impediments include marine pollution, inadequate infrastructure (6), weak policy frameworks, overfishing, climate change, untreated sewage, lack of awareness, and unfair trade practices (6, 7). The cumulative impact of these issues has led to an estimated 80% reduction in Pakistan's fish population (4).

Another area of concern is the excessive use of antibiotics and chemicals to control fish diseases (8). Frequent and misuse of antibiotics is linked to antibiotic and multidrug resistance (MDR) in bacteria inhabiting the gastrointestinal tract (GIT) of fish (9). A study reported two antimicrobial-resistant genes (ARGs)—bla_CTX − M−55_ and QnrVC5—and mutations in the gyrA, gyrB, and parC genes in *Vibrio vulnificus*, a bacterial pathogen isolated from *Lates calcarifer* (Asian sea bass). These genes contributed to the evolution of MDR in this bacterium (10). Another bacterium, *Edwardsiella tarda* isolated from the fishes *Oreochromis niloticus* (Nile Tilapia) and *Clarias gariepinus* (African catfish), exhibited MDR against six classes of antimicrobials: sulfonamides, tetracyclins, aminoglycosides, fluoroquinolones, lincosamides, and penicillin (11). Additionally, bacterium *B. cereus* in *Mugil seheli* (Bluespot mullet) was shown to carry MDR genes *bla1, bla2, tetA*, and *ermA* (12).

This resistance is a threat to aquatic ecosystems and the higher trophic levels of the food chain through the processes of bioaccumulation, biomagnification, and the spread of antibiotic resistance genes (13–15). Recent studies have highlighted the detrimental impact of MDR on aquaculture sustainability, calling for stringent regulations on antibiotic use and the promotion of alternative approaches (16–20). In light of these concerns, sustainable alternatives to antibiotics are urgently needed. Feed supplements, particularly probiotics, prebiotics, and synbiotics, offer a promising, eco-friendly, and cost-effective solution (21–23). These supplements have been reported to contribute to the immune response, resistance to disease, improvement of digestion, growth and survival, protection against pathogens, and better pond water quality in aquaculture (24–26). Two studies documented the positive effects of synbiotics (Lacto Forte), as well as β-1,3-glucan and fructooligosaccharides, on the histological and hematologic profiles, growth, immunity, and antimicrobial resistance in *O. niloticus* and *Litopenaeus vannamei* (Pacific white shrimp), respectively (27, 28).

With reference to aquaculture, probiotics can be defined as the live whole microbes or components of microbes that, when administered either as a feed supplement or rearing water, provide health benefits by improving gut microbiota composition and function (29–31). Their mechanisms of action include competitive exclusion of pathogens, production of inhibitory compounds, enhancement of digestion, stimulation of host immunity, and modulation of quorum sensing (QS) (31). In aquaculture, maintaining a stable microbial ecosystem is essential due to the continuous interaction of farmed fish with their aquatic environment, which influences their health and growth (21, 32).

Several probiotic species have been identified from the gut microbiota of fish, including *Pediococcus acidilactici, P. pentosaceus, Levilactobacillus brevis, Lactobacillus acidophilus, Lactiplantibacillus pentosus*, and *L. plantarum* (33); *Enterococcus* sp., *Weissella cibaria, Lactococcus lactis*, and *Limosilactobacillus fermentum* (34); *Lactococcus garvieae* (35); *Shewanella* sp., *Proteus* sp., and *Alcaligenes* sp. (36); *L. lactis* CLFP 101, *L. plantarum* CLFP 238, and *L. fermentum* CLFP 242 (37).

Common sole or *Solea solea* inhabits muddy and sandy seabeds, coastal areas, and shallow waters and exhibits seasonal migration to deeper waters in winter. It is a nocturnal predator that feeds on small crustaceans and benthic invertebrates (38). In Pakistan, it is a commercially important fish and, along with other marine fishes, constitutes 0.51 million tons of total fisheries production (https://agro.tdap.gov.pk/pakistan-seafood). Being a part of the broader fisheries sector, it contributes to the economy of the country through employment opportunities, export earnings, value-added products, and income generation (39). In Pakistan, due to manufacturing costs, labor, economic pressures, fuel prices, and need and supply dynamics, the price of this fish increased significantly by 2024—for example, in Karachi (1,800 PKR) and in Lahore and Islamabad (789 to 1,579 PKR per pound). Being part of coastal cultural festivities and customs, as well as a selected staple food, it is considered a cultural and culinary treasure of Pakistan. However, the species faces economic difficulties (40). For its preservation, there is a crucial need for sustainable practices like feed improvement with probiotic supplementation.

Recent studies suggest that probiotics application in aquaculture not only enhances fish health as being alternative to antibiotics and vaccines, inhibiting the pathogens' growth, accelerating the resistance to diseases, and improving the digestibility of fats, but also improves water quality by reducing harmful bacterial loads and nitrogenous wastes (24, 41). Additionally, within the context of Pakistan's aquaculture sector, there are several limitations of probiotics, such as the dependency of probiotics' efficacy on regional environmental factors and water quality, poor optimization of already commercialized probiotics, and inaccessibility of smaller fish farms to costly probiotics available in the Pakistani market. Given the cultural importance and economic difficulties associated with *S. solea*, the vital role of probiotics in aquaculture sustainability, and barriers in the successful implementation of probiotics in Pakistan, this study was initiated to identify the native probiotic strains from healthy *S. solea* fish through comprehensive *in vitro* screening strategies.

In addition to biochemical and genomic characterization, the safety and antagonistic properties of these isolates against fish pathogens were evaluated. Following *in vivo* validation, these probiotics could serve as viable alternatives to antibiotics, promoting sustainable and resilient aquaculture practices.

## 2 Materials and methods

### 2.1 Fish guts collection and preparation

*In vitro* characterization was performed at experimental facilities of the School of Biochemistry and Biotechnology (SBB), Punjab University (PU), Lahore. Ten healthy *S. solea* fish, commonly known as sole fish, were bought from a commercial supplier located at Qadimi Shehar, Lahore. Healthy and fresh fish with no apparent pathological symptoms were purchased. Length and weight of fish ranged between 25 and 30 cm and between 170 and 230 g, respectively. These values are consistent with the weight and length reported for healthy *S. solea* fish by IFCA North West and the Food and Agriculture Organization (https://www.nw-ifca.gov.uk/managing-sustainable-fisheries/species/fish/sole) and the Food and Agriculture Organization of the United Nations (https://www.fao.org/fishery/en/culturedspecies/solea_spp/en). Fish specimens that exhibited signs of illness or were in bad condition were not considered for purchase.

For fish handling, transport, and dissection, the Guidelines of CPCSEA for Experimentation on Fishes were followed (42, 43). To minimize the tissue damage, dead fish were handled gently. To prevent contamination with pathogenic bacteria, personal protective equipment (PPE) was used. Specimens were cushioned with sterilized foam and quickly transported in an insulated cooler. Fish were stored in the refrigerator for a few hours. Fish were dissected by a veterinarian under sterile conditions to remove the gut. Fish carcasses were disposed of according to waste disposal protocols (https://www.msdvetmanual.com). Fish guts were thoroughly washed with sterilized distilled water and phosphate buffer saline (PBS) buffer (100 mL) to remove the mucus, grimes, and feed materials (44). The posterior part of the intestine was cut from the cleaned gut and homogenized using sterile glass pestles in autoclaved 100 mL Ringer solution (45). To remove fungal contaminants, the previously reported method of boiling homogenate at 80°C for 15–20 min was employed (46). Following this, the homogenate was incubated at 25°C up to 6 h to facilitate bacterial isolation.

### 2.2 Bacterial isolation and stock preparation

The isolation of bacteria associated with the fish gut was performed using Mueller-Hinton Agar (MHA) medium (47). The gut-homogenized Ringer solution was serially diluted up to 6-fold, and each dilution was spread onto MHA plates. These plates were incubated at 37°C for 24 h to allow bacterial growth (48). The following day, bacterial colonies were analyzed, and the colony-forming units (CFU) per mL were calculated using the following formula:


$$\begin{array}{l} \frac{CFU}{ml}=\;\frac{No.\;of\;colonies\;\times Dilution\;factor\;}{Volume\;of\;culture} \end{array}$$

The streak plate method was used to isolate purified colonies, which were then stored in 50% glycerol stock solution at −20°C for long-term preservation (49). Bacterial morphological analysis was performed by examining the shape, color, size, and texture of the colonies.

### 2.3 Biochemical characterization

To characterize the bacterial isolates biochemically, several tests were conducted, including the catalase test, mannitol fermentation test, glucose, lactose, and fructose fermentation tests, as well as assays for hydrogen cyanide (HCN) production, cellulose degradation, chitinase, and pectinase production. For the catalase test, a freshly grown overnight bacterial culture was used to prepare the smear. A few drops of hydrogen peroxide were then added to the fixed smear, and the results were compared with a negative control.

For the mannitol fermentation test, autoclaved medium was used as a substrate to assess the ability of the isolates to ferment mannitol sugar. An overnight culture was streaked onto MSA plates and incubated at 37°C for 24 h. After incubation, color changes in the medium were observed and compared with a negative control (50).

For the glucose fermentation test, 5 mL of syringe-filtered glucose medium was added to a sterile test tube and inoculated with 20 μL of freshly grown overnight culture. The culture was incubated overnight at 37°C in a shaking incubator (150 rpm), and the color change in the medium was recorded and compared with a negative control. For the sucrose fermentation test, 5 mL of syringe-filtered sucrose medium was used as a substrate in a sterile test tube. The medium was inoculated with 20 μL of freshly grown overnight culture and incubated overnight at 37°C in a shaking incubator (150 rpm). A color change in the medium indicated the bacterial ability to ferment sucrose, with results compared with a negative control (51).

For the lactose fermentation test, 5 mL of syringe-filtered lactose medium was inoculated with 20 μL of freshly grown overnight culture, incubated at 37°C in a shaking incubator (150 rpm), and the color change in the medium was recorded and compared with a negative control (51).

For the HCN production test, 25 mL of sterile glycine agar medium was poured into Petri plates. Once solidified, a freshly grown overnight culture was spread onto the agar surface. Autoclaved filter paper dipped in picric acid solution (used as an indicator) was placed on a sterile Petri plate lid, and the plate was sealed with parafilm. The plates were incubated at 37°C for 48 h. A color change in the filter paper indicated HCN production, while no change indicated the absence of HCN. The results were compared with a negative control (52).

For the cellulase production test, autoclaved carboxy-methyl cellulose (CMC) medium was used. Autoclaved solidified media plates were prepared, and agar wells were formed. A synchronized overnight culture was inoculated into these wells and incubated at 37°C for 24 h. The zone of inhibition was assessed using iodine solution as an indicator, and the results were compared with a negative control (53).

For the chitinase production test, autoclaved chitin medium was used. Solidified agar plates were prepared, and agar wells were formed. A synchronized overnight culture was introduced into these wells and incubated at 37°C for 24 h. The zone of inhibition was determined using iodine solution, and the results were compared with a negative control. For the pectinase production test, autoclaved pectin media was used. Similar to the previous assays, solidified agar plates were prepared, and agar wells were formed. A synchronized overnight bacterial culture was poured into these wells and incubated at 37°C for 24 h. The zone of inhibition was checked using iodine solution, and the results were compared with a negative control (54).

### 2.4 Molecular characterization

The organic method was used for DNA extraction by following a previously documented protocol. This method involved the use of phenol:chloroform:isoamyl alcohol (55). DNA integrity was confirmed by agarose gel electrophoresis at 90 V for 45 min. Afterward, gel visualization was performed in a gel documentation system under UV. DNA was stored for future use at −20°C (56).

Polymerase chain reaction (PCR) was performed to amplify the 16S rRNA gene in isolated DNA. Previously documented 16S rRNA gene-specific primers were used (57). Sequences, melting temperatures (Tm), and GC content (the proportion of guanine and cytosine bases) of forward and reverse primers were AGAGTTTGATCCTGGCTCAG, 55°C, and 50% and AAGGAGGTGATCCAGCCGCA, 60°C, and 50%, respectively. The PCR amplicon size was 1500 bp. The PCR reaction mixture (25 μL) was prepared using 0.5 μL Template DNA, 1 μL forward primer, 1 μL reverse primer, 12.5 μL PCR master mix (DNA polymerase, dNTPs, MgCl_2_, and PCR buffer), 9.5 μL PCR water, and 0.5 μL Taq polymerase. All these components were added to the PCR tube by placing the tube in an ice bucket. PCR reaction was carried out under optimized conditions: initial denaturation (95°C for 5 min), cyclic denaturation (95°C for 45 seconds), annealing (58°C for 40 seconds), and cyclic extension (92°C for 1 min and final extension (72°C for 10 min). The number of cycles was 35X (58).

DNA purification from post-PCR agarose gel bands was performed using the FavorPrep^TM^ gel purification mini kit (Cat # FAGCK 001). The purified DNA samples were then shipped to Macrogen, Korea, for Sanger sequencing analysis.

Sequencing results were obtained in the FASTA format. On the NCBI platform, the basic local alignment search tool (BLAST) was used to determine the similarity index between the obtained bacterial sequences and existing bacterial species in the database. Multiple sequence alignment was performed using the Clustal Omega Multiple Sequence Alignment Tool (https://www.genome.jp/tools-bin/clustalw) (59). Gaps were removed from aligned sequences. Ungapped sequences were used to construct the phylogenetic tree. To construct a phylogenetic representation of evolutionary relationships, the neighbor-joining statistical method was used. The lengths of the branches in the tree directly represented comparisons between different sequences at the genetic level.

Bacterial DNA sequences were submitted to the publicly accessible NCBI GenBank database to obtain the assigned accession numbers (60). The accession numbers assigned to five bacteria, *L. rhamnosus* SBBPro6, *E. faecium* SBBPro7, *B. amyloliquefaciens* SBBPro8, *B. subtilis* SBBPro9, and *B. cereus* SBBPro10, were PQ002180, PQ002492, PQ002184, PQ002187, and PQ002188, respectively.

### 2.5 Growth curve analysis

Bacterial growth analysis was performed in triplicate. For this analysis, synchronized cultures with OD^600^ = 0.1 were prepared and used to inoculate the medium. The control consisted of uninoculated medium. Initially, the OD^600^ of the control and experimental tubes was measured at 0 h. Following this, tubes were incubated (37°C and 150 rpm), and OD600 was measured at different time intervals: 3, 6, 24, 27, 30, 48, 51, and 54 h. Time was plotted against OD^600^ to generate the growth curve (61).

### 2.6 Probiotic assays

#### 2.6.1 Bile salt tolerance assay

Autoclaved De Man-Rogosa-Sharpe (MRS) broth supplemented with 0.3 g bile salt (Himedia, Cat # RM008-500G) was inoculated with the isolates from this study. Control contained MRS and inoculum without bile salts. Following this, bacteria were cultured at 37°C and 150 rpm until the exponential phase was achieved. Then, OD^600^ of the culture was measured at 0 and 24 h. A bar graph was plotted to compare the growth of bacteria in the presence of bile salts, which reflected the bacterial resistance against bile salts (62).

#### 2.6.2 NaCl tolerance assay

Bacteria were grown for 24 h to prepare the synchronized cultures. Culture of each bacterium with OD^600^ = 1 was used to inoculate three MRS media containing different NaCl (WEL GENE Precision Solution^TM^, Cat # ML 011-01) concentrations: 0.2%, 2%, and 5%. Afterward, OD^600^ was measured at 0 h. All test tubes were then kept in a shaking incubator (37°C and 150 rpm) for efficient growth. After 24 h, OD^600^ was measured again to check the NaCl tolerance potential of isolates (63).

#### 2.6.3 pH tolerance assay

MRS media adjusted at pH values 2, 3, and 5 using 1N HCl and 1N NaOH were prepared. Overnight-grown cultures with OD^600^ = 1 were inoculated into the medium to synchronize the cultures. A negative control, MRS medium without any inoculum, was used. The OD^600^ of control and experimental cultures was measured at 0 h. All test tubes were then kept in a shaking incubator (37°C and 150 rpm) for efficient growth. After 24 h, OD^600^ was assessed again to check the growth of isolates at different pH values (64). Bar graphs were plotted to analyze and compare the growth potential of bacteria at different pH levels.

#### 2.6.4 Survival potential of isolates in simulated gastric conditions

To investigate the survival potential of the isolates from this study under gastric conditions, simulated gastric medium was synthesized. Previously reported composition of simulated gastric fluid was used with slight modifications: KCl (0.0256 g), KH_2_PO_4_ (0.0061 g), NaHCO_3_ (0.105 g), NaCl (0.1375 g), MgCl_2_.(H_2_O)_6_ (0.0012 g), (NH_4_)_2_CO_3_ (0.0024 g), HCl (0.0284 g), and CaCl_2_ (H_2_O)_2_ (0.0011g) per 50 mL of distilled water, and filtered through a microporous membrane (0.2 μm pore size) (65). The 15 mL of simulated gastric fluid was supplemented with pepsin solution (3.2 mL), 0.3M CaCl_2_ (0.01 mL), and distilled water (1.8 mL). The pH was adjusted to 2. Overnight-grown fresh bacterial cultures were centrifuged at 6,000 × g, 4°C for 20 min to pellet down the cells. Following this, cells were suspended in 0.9% saline solution (1 mL). Saline solution was used as an alternative to broth. The broth (1 mL) was inoculated into gastric medium (9 mL) and incubated for 3 h under shaking conditions (150 rpm). After 3 h, each isolate sample was taken from the respective tube and streaked on MHA agar plates, followed by incubation at 37°C for 24 h. Growth of bacteria was observed (66).

#### 2.6.5 Cell adhesion assay

To remove the surface mucous, the ileum was washed in PBS for 30 min at 4°C. Following this, small pieces of ~1 cm of the ileum were cut and placed in an overnight-grown bacterial cell suspension for different time intervals: 30, 60, and 90 min. These pieces were macerated and seeded on freshly prepared autoclaved MHA agar plates. Afterward, plates were incubated at 37°C for 24 h. Growth was observed through enumerating CFUs of each isolate at different time intervals (67).

#### 2.6.6 Hemolytic assay

Blood agar was prepared using peptone (0.5%), yeast or beef extract (1.5%), agar (1.5%), NaCl (0.5%), distilled water, and sheep blood (5%). The medium was autoclaved before the addition of blood. Blood agar was poured into the Petri plates and solidified. Each isolate was streaked on a blood agar media plate and incubated at 37°C for 24 h. Following this, the hemolytic activity/type (α, β, and ⋎) of each isolate was observed (68).

#### 2.6.7 Antimicrobial test

The antibacterial potential of the isolates from this study was assessed using *Pseudomonas aeruginosa* and *Staphylococcus aureus*. Overnight culture (200 μL) of each strain was added to a freshly prepared MRS media plate using the spread plate method (69). Freshly grown pathogenic culture was added to wells made in solidified MRS agar. All plates were then kept in an incubator at 37°C for 24 h (70). After the incubation, zones of inhibition and colony formation around the wells were observed and recorded to assess the antibacterial potential of each fish gut-borne bacterium against the pathogens used.

#### 2.6.8 Heat shock tolerance assay

Bacterial cells were pelleted down by centrifugation at 4,000 rpm at 4°C for 5–7 min, washed with 1 mL of PBS solution, and subjected to heat shock at 100°C for 2–3 min. Pellet was suspended in growth medium (5 mL) and incubated at 37°C and 150 rpm overnight. OD^600^ of each strain was measured to assess the heat shock tolerance potential of bacteria (71).

#### 2.6.9 Cholesterol assimilation test

Isolates properties of cholesterol breakdown were checked by cholesterol liquicolor kit (PATHOZYMES DIAGNOSTICS, India) using the CHOD-PAP method (72). Cholesterol standard (10 μL) was added to a cuvette containing 1 mL of culture. Mixed thoroughly and incubated for 5 min at 20–25°C. A cuvette containing a cholesterol standard without culture was used as a standard. Following incubation, the absorbance at 505 nm (OD^505^) was measured. The cholesterol concentration of standard and test samples was estimated using the following formula:


$$\begin{array}{r} Cholesterol\;conc.\;of\;standard(mg/dl)\\ =553\;x\;Absorbance\;value\;of\;standard\\ Cholesterol\;conc.\;of\;test\;sample\;(\frac{mg}{dl})\\ =\;\frac{Absorbance\;value\;of\;test\;sample}{Absorbance\;value\;of\;standard}\times 200 \end{array}$$

#### 2.6.10 Antibiotic sensitivity test

Different antibiotics—erythromycin (250 mg), azithromycin (250 mg), ciprofloxacin (500 mg), amoxil (500 mg), and ampicillin (250 mg)—were used to perform the antibiotic sensitivity profiling of the isolates. The disk diffusion method was used to perform this test (73). Disks were prepared manually using dried Whatman filter paper no. 3. Antibiotic stock solutions were prepared, added to disks in a predefined amount, and allowed to absorb for 10 min. Disks were dried in an oven for 15 min at 37°C. MRS agar plates were prepared and inoculated with an overnight-grown fresh culture of bacteria via spreading. Then, antibiotic disks were evenly placed on the plates using sterilized forceps. Different dilutions of antibiotics (10, 20, and 30 μL) were used to assess the extent of sensitivity. The effect of antibiotics on the isolates was determined by measuring the diameter of the zones of inhibition (mm). The zone size indicated the bacterial susceptibility against antibiotics.

### 2.7 Statistical analysis

Each experiment was performed in triplicate, and the results were represented as mean ± SD. To evaluate the probiotic potential of the five isolates, statistical analyses were conducted using SPSS version 20 (74). One-way ANOVA was performed to determine the significance status of the *p*-value (75). Dunnett's test was performed for NaCl and bile salt tolerance tests and temperature and pH resistance tests to determine whether the growth of each probiotic strain significantly differed from the control group. Dunnett's test was specifically chosen as a *post hoc* analysis to compare each probiotic strain directly with the control, rather than comparing the probiotics with each other (76). For the antibiotic tolerance test and cell adhesion test, a one-sample *t*-test was applied (77). As the control group had a value of zero, it was not suitable to apply the one-way ANOVA and Dunnett's test. Based on the statistical analyses, significant differences in probiotic performance were accurately identified.

## 3 Results

### 3.1 Bacterial isolation

Bacterial colonies were observed on an MHA agar plate after 24 h of incubation. CFUs of isolates were calculated as follows: *Lacticaseibacillus rhamnosus* SBBPro6 and *Enterococcus faecium* SBBPro7 (19 × 10^−4^ per mL), *Bacillus amyloliquefaciens* SBBPro8 and *Bacillus subtilis* SBBPro9 (36 × 10^−5^ per mL), and *Bacillus cereus* SBBPro10 (11 × 10^−6^ per mL) (Supplementary Figure S1).

### 3.2 Morphological analysis

Isolates were examined through colony features such as texture, shape, color, margins, and elevation. All colonies showed off-white color with creamy texture. Gram-positive character, spindle shape, full margins, and raised elevation were exhibited by *L. rhamnosus* SBBPro6 and *B. amyloliquefaciens* SBBPro8. Irregular shape, lobate margins, and raised elevation were observed in *E. faecium* SBBPro7. *B. subtilis* SBBPro9 and *B. cereus* SBBPro10 also showed Gram-positive features with rod shape, entire margins, and convex elevation (Supplementary Figure S2).

### 3.3 Biochemical characterization

In catalase production activity, instant effervescence was recorded in all isolates, which reflected their antioxidant property (Supplementary Figure S3). In the mannitol fermentation test, *L. rhamnosus* SBBPro6, *E. faecium* SBBPro7, and *B. amyloliquefaciens* SBBPro8 showed maximum potential to ferment mannitol, as the media color changed from pink to yellow, while *B. subtilis* SBBPro9 showed no growth and *B. cereus* SBBPro10 exhibited mild growth (Supplementary Figure S4). Each isolate exhibited the potential to ferment glucose, lactose, and fructose (Supplementary Figure S5).

All isolates were positive for HCN, cellulase, and chitinase production tests (Supplementary Figures S6–S8). All bacteria were negative for the pectinase production test (Supplementary Figure S9, Supplementary Table S1).

### 3.4 Molecular characterization and phylogenetic tree construction

Extracted DNA and PCR amplicons were visualized on a gel to check their quality (Supplementary Figures S10, S11). FASTA sequences were submitted to GenBank and assigned the following accession numbers: *L. rhamnosus* SBBPro6 (PQ002180), *E. faecium* SBBPro7 (PQ002492), *B. amyloliquefaciens* SBBPro8 (PQ002184), *B. subtilis* SBBPro9 (PQ002187), and *B. cereus* SBBPro10 (PQ002188).

**Figure 1:**
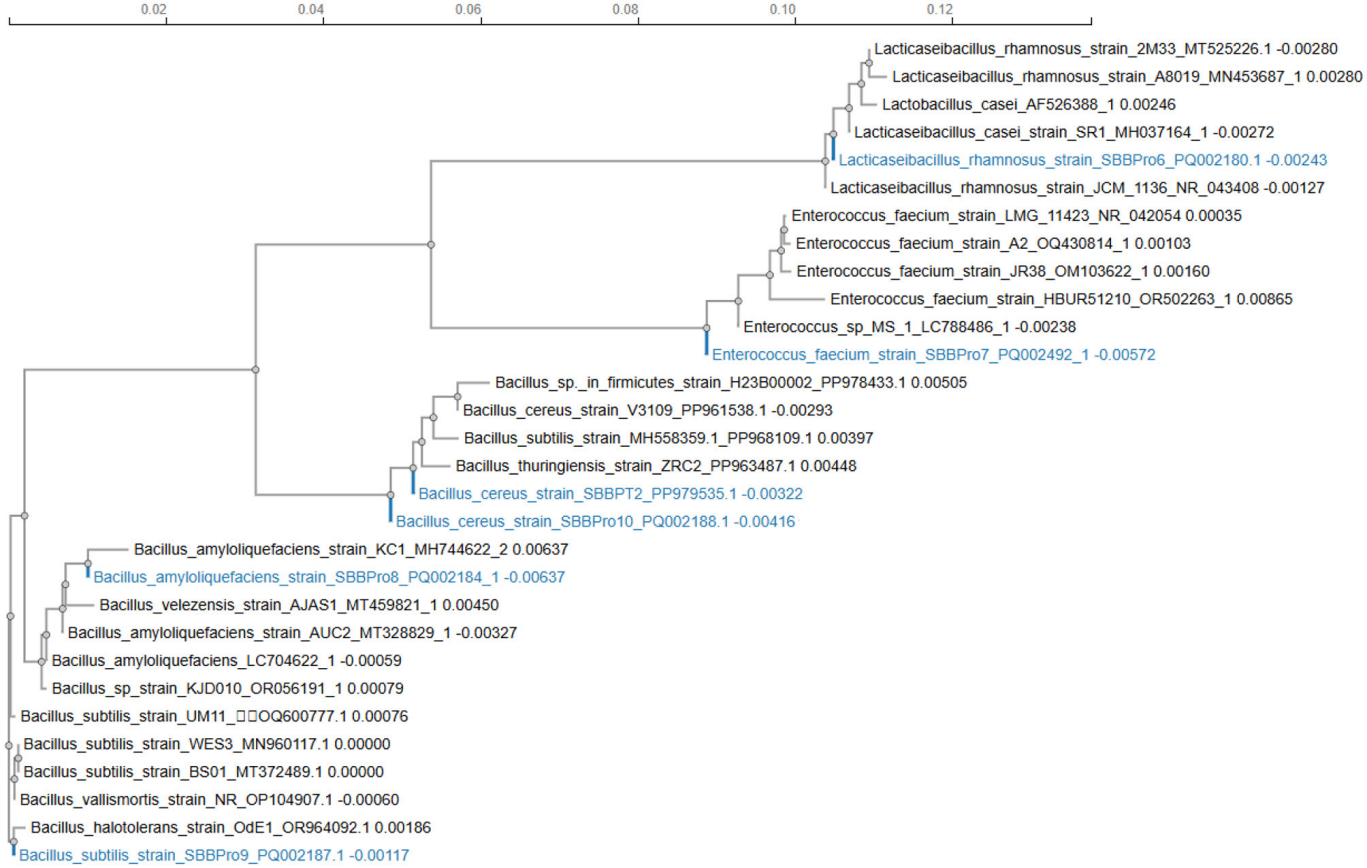
Phylogenetic tree construction for bacteria isolated in the current study using the neighbor-joining statistical method through the ClustalW package.

The phylogram displayed *Lacticaseibacillus rhamnosus strain SBBPro6, L. rhamnosus*, and *L. casei* strains as a cohesive group with minimal phylogenetic distinctions among them. The phylogenetic analysis confirmed that *L. rhamnosus* SBBPro6 fits perfectly into the *Lacticaseibacillus* genus, making it a prototypical representative. The position of *Enterococcus faecium strain SBBPro7* showed complete affiliation with a group consisting of *E. faecium* strains exclusively. The close position to strains LMG_11423 and JR38 demonstrated evolutionary continuity. Genetic diversity in this clade is moderate. Strain *Bacillus amyloliquefaciens SBBPro8* formed a tight cluster with additional strains of its species, such as KC1 and AUC2, because of their short branch lengths, which suggested similarities in their genetic sequences. This tight clustering demonstrated evolutionary conservation. *Bacillus subtilis strain SBBPro9* belonged to the *B. subtilis* complex, where it stood nearest to strains BS01 and WES3. The genetic makeup of SBBPro9 showed only moderate variation from its genus specifications, yet it still stayed cohesive with its genus groups. The two *Bacillus cereus* strains SBBPro10 and SBBPT2 existed as members of the extensive *B. cereus phylogenetic* group. Genetic variations within *B. cereus* appeared to affect the branch length of SBBPro10 compared to SBBPT2, as the second strain exhibited more similarity to other *B. cereus* strains. Their near position demonstrated shared evolutionary beginnings, although they could exhibit distinct characteristics at the strain level for functional or ecological behavior (Figure 1).

### 3.5 Growth curve study

**Figure 2:**
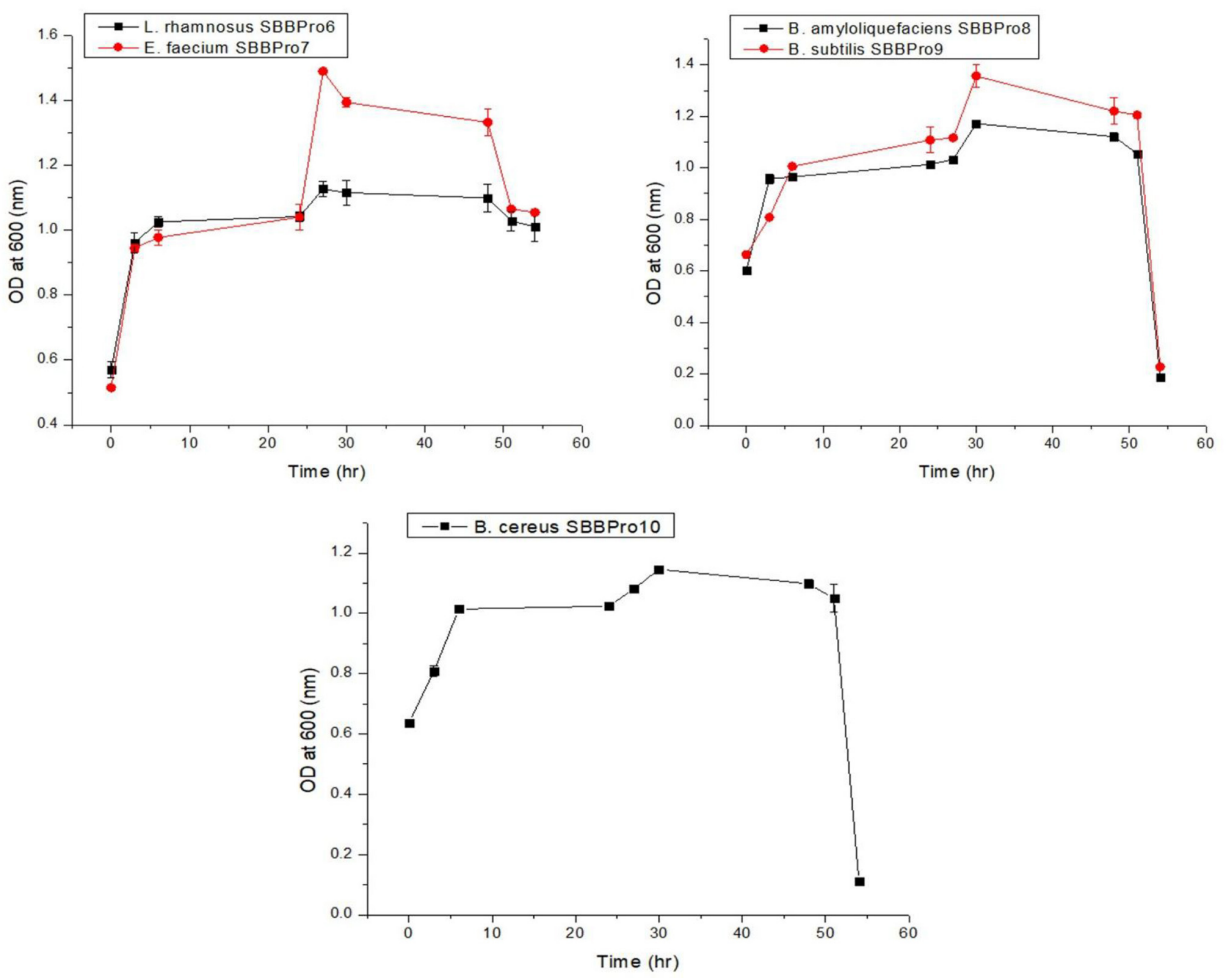
Growth curves of bacteria isolated in the current project obtained by plotting time vs. OD^600^.

Each isolate exhibited an efficient growth rate. *L. rhamnosus* SBBPro6 and *E. faecium* SBBPro7 were the fastest growing, with the log phase starting at 27 h, as both of these showed optimum OD^600^ at this time interval, i.e., 1.127 ± 0.024 and 1.490 ± 0.003, respectively. In *B. amyloliquefaciens* SBBPro8, *B. subtilis* SBBPro9, and *B. cereus* SBBPro10, the maximum growth was recorded at 30 h with OD^600^ = 1.172 ± 0.008, 1.356 ± 0.008, and 1.146 ± 0.01, respectively (Supplementary Table S2, Figure 2).

### 3.6 Bacterial characterization through probiotic assays

#### 3.6.1 Bile salt tolerance assay

**Figure 3:**
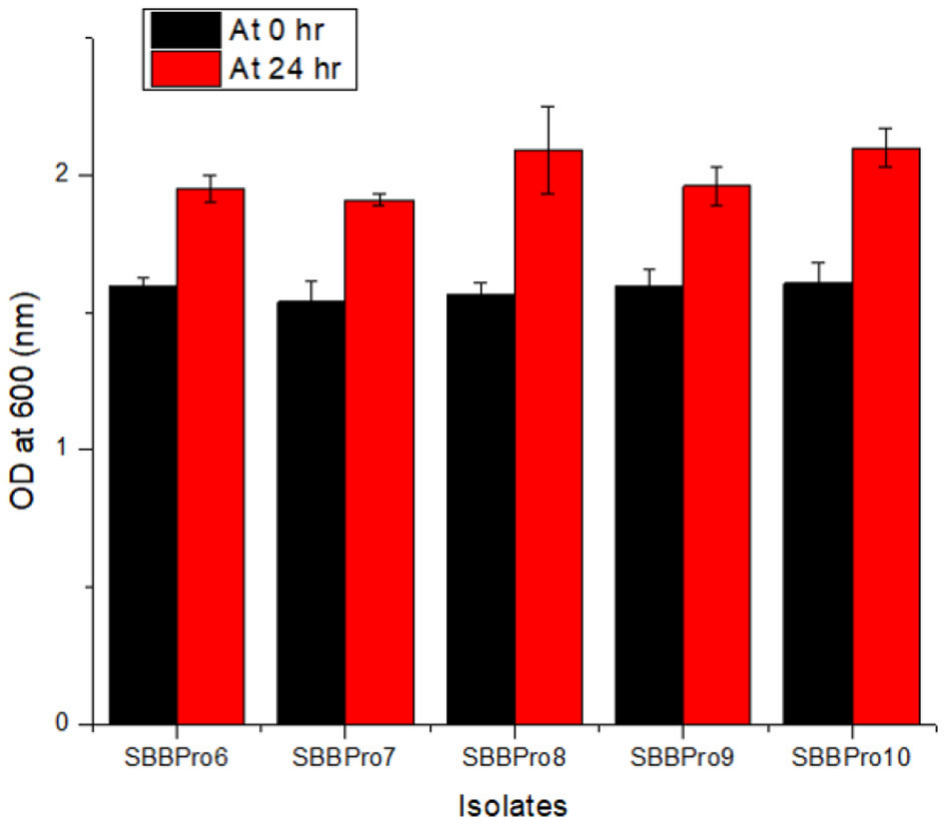
Bile salt tolerance assay of bacteria isolated in the current study performed at 0.3% bile salt concentration: *L. rhamnosus* SBBPro6, *E. faecium* SBBPro7, *B. amyloliquefaciens* SBBPro8, *B. subtilis* SBBPro9, and *B. cereus* SBBPro10.

The isolates from this study showed maximum tolerance for bile salts. Among all the isolates, *B. cereus* SBBPro10 showed maximum tolerance against bile salt with the highest OD^600^, i.e., 2.10 ± 0.07, while *E. faecium* SBBPro7 exhibited minimum growth with the lowest OD^600^, i.e., 1.91 ± 0.02. The results were found significant with a *p*-value < 0.05 (Supplementary Tables S3, S4, Figure 3).

#### 3.6.2 NaCl tolerance assay

**Figure 4:**
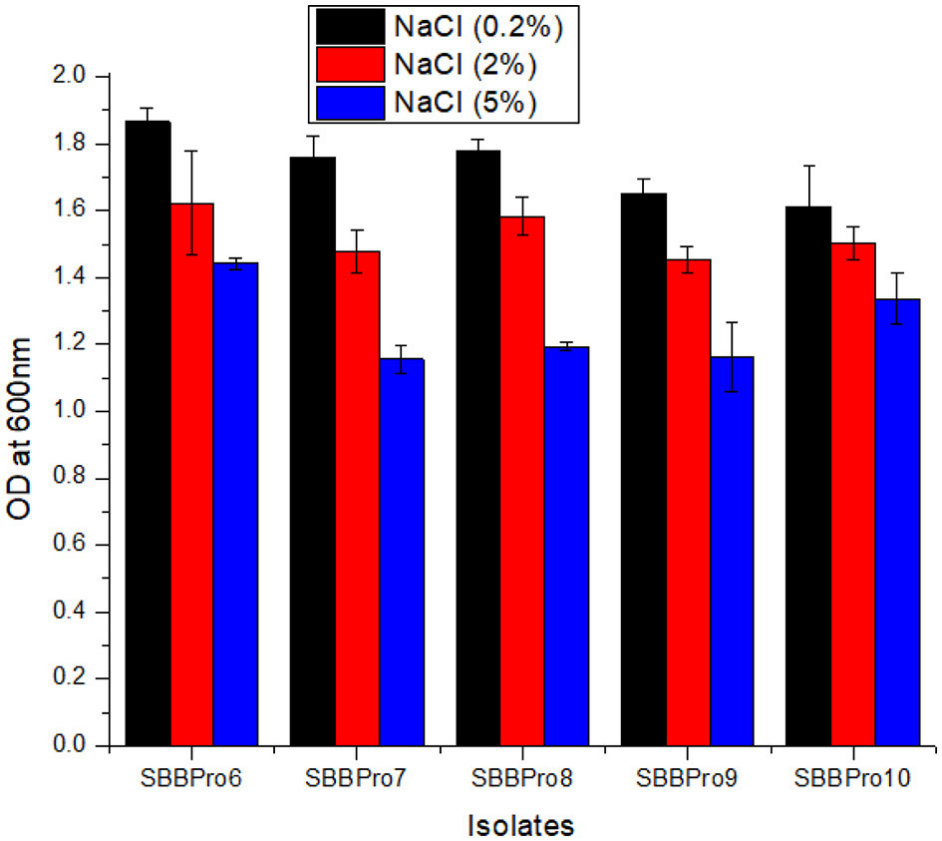
Growth of isolated bacteria at 0.2%, 2%, and 5% concentrations of NaCl analyzed through measurement of OD^600^ at logarithmic phase: *L. rhamnosus* SBBPro6, *E. faecium* SBBPro7, *B. amyloliquefaciens* SBBPro8, *B. subtilis* SBBPro9, and *B. cereus* SBBPro10.

The growth of bacteria was found to drop slightly with an increase in NaCl concentration. In all the isolates from this study, OD^600^ was highest at 0.2%, followed by 2%. At 0.2%, the OD^600^ was highest (1.86 ± 0.04) in *L. rhamnosus* strain SBBPro6 and lowest (1.61 ± 0.11) in *B. cereus* SBBPro10. At 2% concentration, maximum and minimum OD^600^ values were observed in *L. rhamnosus* SBBPro6 (1.62 ± 0.15) and *B. subtilis* SBBPro9 (1.45 ± 0.03), respectively. At 5% concentration, maximum OD^600^ was observed in *L. rhamnosus* SBBPro6, i.e., 1.44 ± 0.01, and minimum was observed in *E. faecium* SBBPro7, i.e., 1.15 ± 0.04. The results were significant with a *p*-value < 0.05 (Supplementary Tables S5, S6, Figure 4).

#### 3.6.3 pH tolerance assay

**Figure 5:**
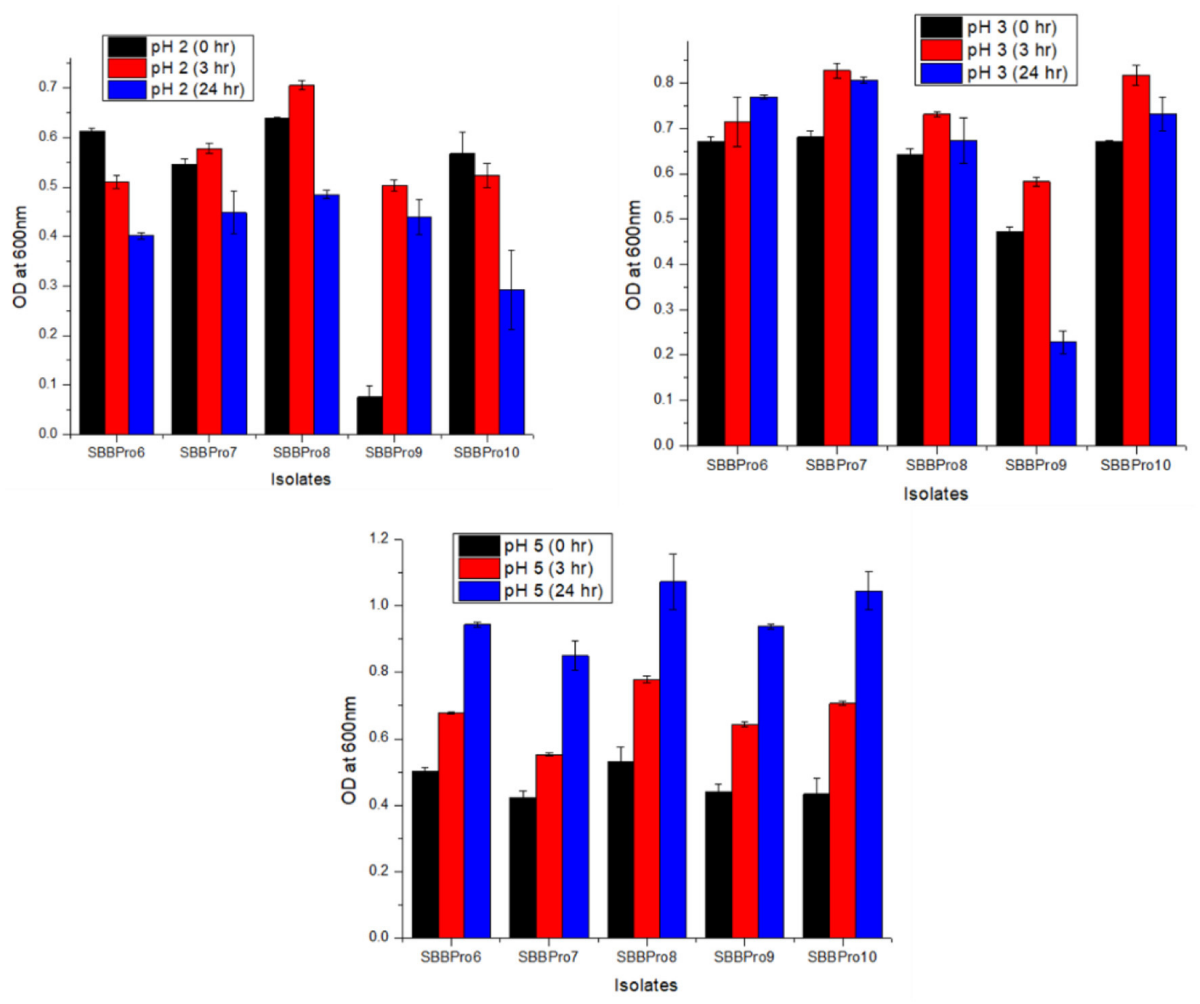
Assessment of pH tolerance potential of bacteria isolated in the current study, at pH values 2, 3, and 5: *L. rhamnosus* SBBPro6, *E. faecium* SBBPro7, *B. amyloliquefaciens* SBBPro8, *B. subtilis* SBBPro9, and *B. cereus* SBBPro10.

The isolates from this study exhibited relatively efficient growth at pH 5, followed by pH 3. Highest OD^600^ values were observed in *B. amyloliquefaciens* SBBPro8 (0.48 ± 0.009) and *B. cereus* SBBPro10 (0.84 ± 0.019) at pH = 2 and 3, respectively. At pH = 5, OD^600^ was observed in the range of 0.85 ± 0.044 and 1.07 ± 0.082. The highest was recorded in *B. amyloliquefaciens* SBBPro8 and the lowest in *E. faecium* SBBPro7, respectively. The results were significant with *p*-values recorded as follows: pH 2 (0.002973), pH 3 (0.000003), and pH 5 (0.001647). At pH 2 and 3, Dennett's test indicated that all probiotic strains showed significantly higher tolerance than the control, with *B. cereus* SBBPro10 demonstrating the highest mean difference at pH = 2 (Supplementary Tables S7, S8a-f, Figure 5).

#### 3.6.4 Simulated gastric conditions survivability assay

All isolates exhibited growth on synthetic gastric fluid media, which showed their potential to tolerate gastrointestinal (GI) conditions of the host body. This confirmed their safety profile as probiotics (Supplementary Figure S12).

#### 3.6.5 Cell adhesion test

**Figure 6:**
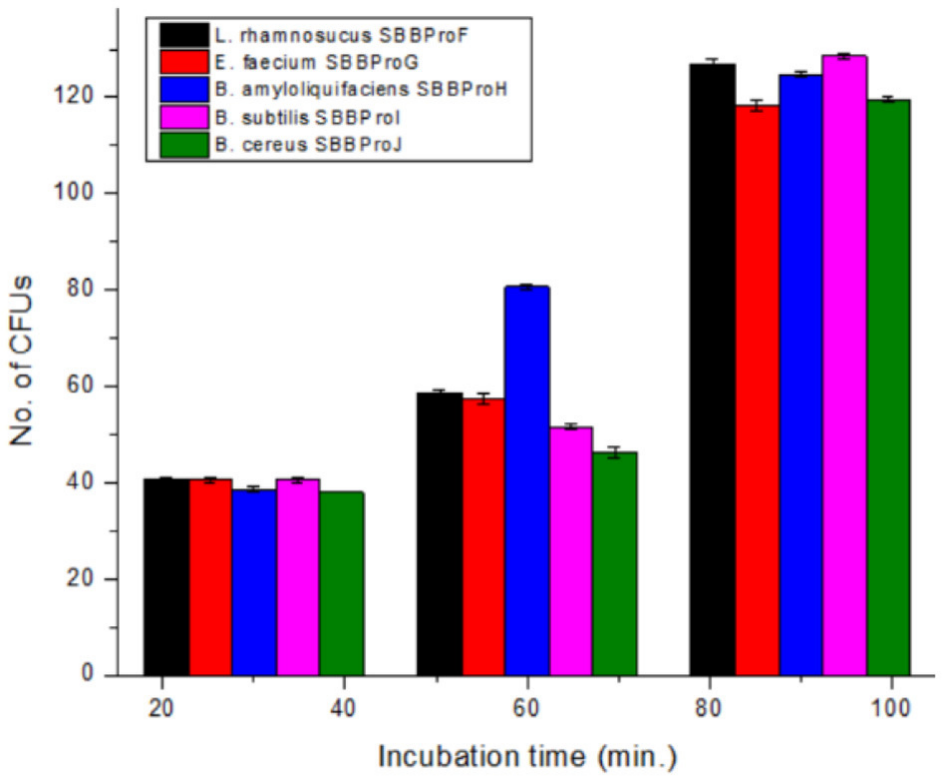
Assessment of cell adhesion potential of bacteria isolated in the current study by cell adhesion assay performed at 30, 60, and 90 min.

Each isolate exhibited the capability of adhesion to epithelial cells of the ileum, and CFUs increased with the incubation time from 30 to 90 min. After 30 min of incubation, *L. rhamnosus* SBBPro6 and *B. subtilis* SBBPro9 showed the maximum CFUs, with a count of 41, while *E. faecium* SBBPro7, *B. amyloliquefaciens* SBBPro8, and *B. cereus* SBBPro10 showed CFUs of 40, 39, and 38, respectively. At 60 min of incubation, *B. amyloliquefaciens* SBBPro8 showed the maximum CFUs of 81, while *L. rhamnosus* SBBPro6, *E. faecium* SBBPro7, *B. subtilis* SBBPro9, and *B. cereus* SBBPro10 showed CFUs of 59, 57, 52, and 47, respectively. At 90 min of incubation, *B. subtilis* SBBPro9 showed CFUs of 129, while *L. rhamnosus* SBBPro6, *B. amyloliquefaciens* SBBPro8, *B. cereus* SBBPro10, and *E. faecium* SBBPro7 showed CFUs of 128, 125, 120, and 119, respectively (Supplementary Figure S13, Table 1, Figure 6). All the isolates demonstrated high cell adhesion efficiency, with a significant *p*-value of < 0.05 (Supplementary Tables S9a–c).

**Table 1:** Assessment of adhesion potential of fish gut-borne bacteria with intestinal cells at three different time intervals.

**Isolates**	**30 min**					**60 min**					**90 min**				
	**I**	**II**	**III**	**Mean**	**SD**	**I**	**II**	**III**	**Mean**	**SD**	**I**	**II**	**III**	**Mean**	**SD**
*Lacticaseibacillus rhamnosus* SBBPro6	40	41	41	40.66	±0.577	59	58	59	58.66	±0.577	128	126	127	127	±1
*Enterococcus faecium* SBBPro7	40	41	41	40.66	±0.577	57	57	59	57.66	±1.154	119	117	119	118.33	±1.154
*Bacillus amyloliquefaciens* SBBPro8	38	39	39	38.66	±0.577	80	81	81	80.66	±0.577	125	124	125	124.66	±0.577
*Bacillus subtilis* SBBPro9	40	41	41	40.66	±0.577	52	51	52	51.66	±0.577	129	129	128	128.66	±0.577
*Bacillus cereus* SBBPro10	38	38	38	38	0	45	47	47	46.33	±1.154	120	119	120	119.66	±0.577

#### 3.6.6 Hemolytic assay

All bacterial isolates showed gamma (γ) hemolysis, i.e., no zone was observed around colonies, which confirmed the safety profile of probiotics (Supplementary Figure S14).

#### 3.6.7 Antimicrobial resistance assay

Among the isolates from this study, *L. rhamnosus* SBBPro6, *E. faecium* SBBPro7, *B. amyloliquefaciens* SBBPro8, and *B. subtilis* SBBPro9 showed resistance against *S. aureus*. In case of *P. aeruginosa, L. rhamnosus* SBBPro6, and *B. subtilis* SBBPro9 were slightly sensitive (Supplementary Figure S15).

#### 3.6.8 Heat shock tolerance assay

**Figure 7:**
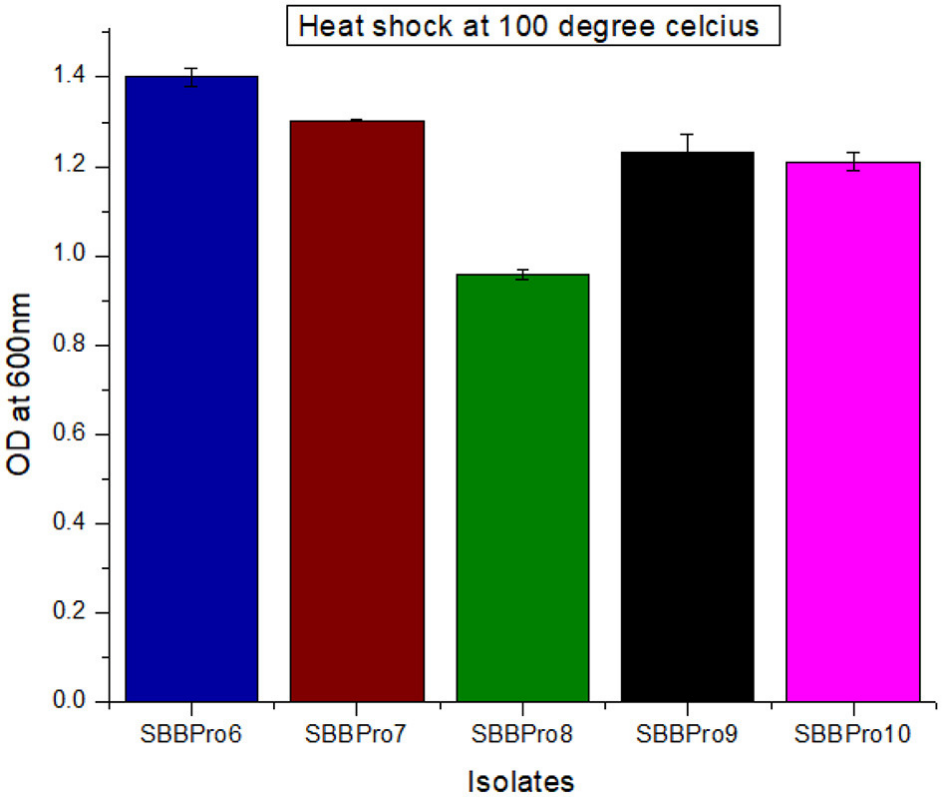
Assessment of heat shock tolerance of bacteria isolated in the current study through measurement of OD^600^ at log phase after heat shock treatment at 100°C.

All isolates survived the heat shock treatment. *L. rhamnosus* SBBPro6 showed maximum growth with OD^600^ = 1.40 ± 0.02, and *B. amyloliquefaciens* SBBPro8 exhibited minimum growth with an OD^600^ value of 0.95 ± 0.01. The results were significant with a *p*-value < 0.05 (Supplementary Tables S10, S11, Figure 7).

#### 3.6.9 Assessment of cholesterol assimilation potential

**Figure 8:**
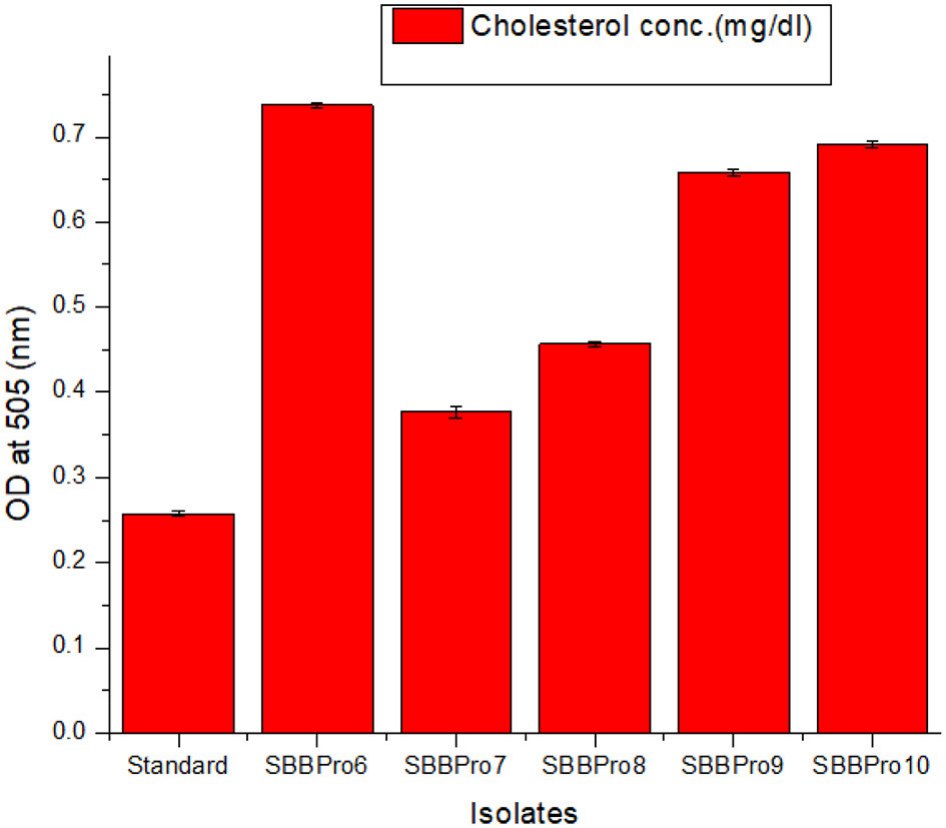
Comparison of cholesterol assimilation efficiencies of the current study documented bacteria through measurement of OD^505^ of standard (STD) and test samples: *L. rhamnosus* SBBPro6, *E. faecium* SBBPro7, *B. amyloliquefaciens* SBBPro8, *B. subtilis* SBBPro9, and *B. cereus* SBBPro10.

All isolates were capable of cholesterol degradation. Compared with the standard, maximum cholesterol degradation was observed in *E. faecium* SBBPro7, i.e., 83.44 mg/dL. Minimum cholesterol degradation was exhibited by *L. rhamnosus* SBBPro6, i.e., 26.768 mg/dL (Table 2, Figure 8).

**Table 2:** Estimation of cholesterol assimilation efficiencies of the current study bacteria based on measurement of OD^505^ through cholesterol liquicolor kit using CHOD-PAP method.

**Isolates**	**OD^505^**	**Cholesterol conc. (mg/dL)**
Standard	0.256	141.568
*Lacticaseibacillus rhamnosus* SBBPro6	0.735	114.8
*Enterococcus faecium* SBBPro7	0.372	58.12
*Bacillus amyloliquefaciens* SBBPro8	0.454	70.9
*Bacillus subtilis* SBBPro9	0.656	102.5
*Bacillus cereus* SBBPro10	0.688	107.5

#### 3.6.10 Antibiotic sensitivity test

**Figure 9:**
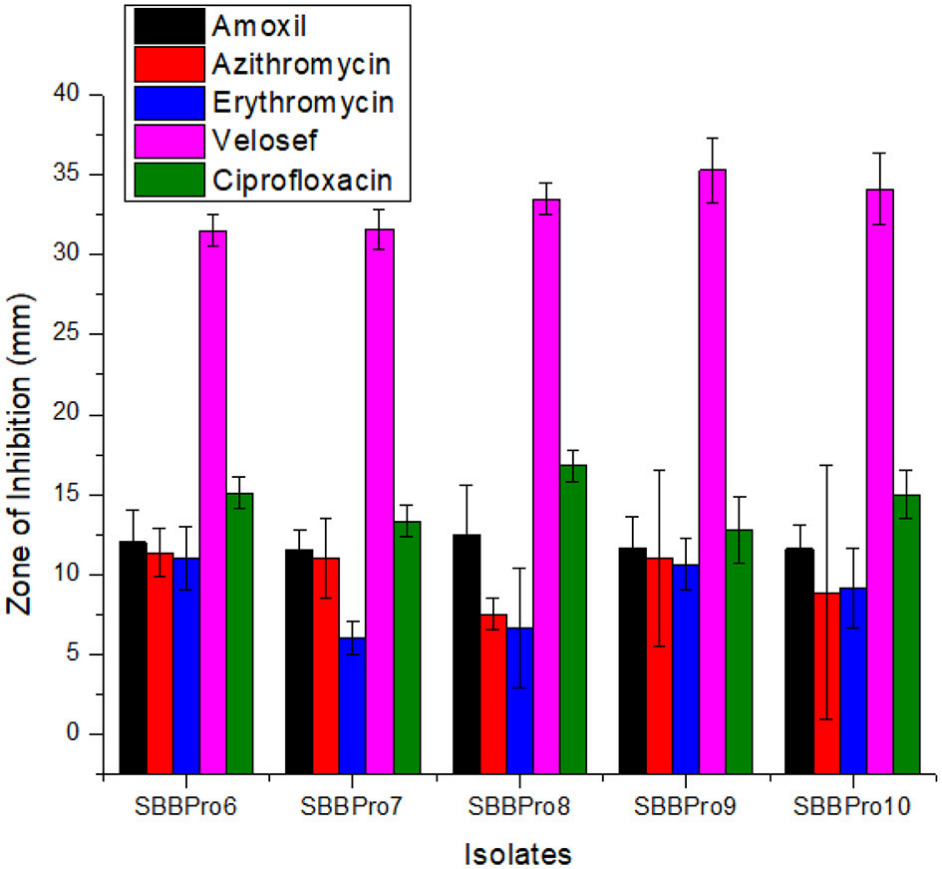
Antibiotic sensitivity profiling of bacteria isolated in the current study against antibiotics amoxil, azithromycin, erythromycin, Velosef, and ciprofloxacin through measurement of zones of inhibition: *L. rhamnosus* SBBPro6, *E. faecium* SBBPro7, *B. amyloliquefaciens* SBBPro8, *B. subtilis* SBBPro9, and *B. cereus* SBBPro10.

Each isolated strain was efficiently sensitive to antibiotics and showed inhibition zones. All of them showed the highest inhibitory zones against Velosef, measuring 31.5, 31.6, 33.5, 35.3, and 34 mm, respectively. In case of amoxil and ciprofloxacin, *B. amyloliquefaciens* SBBPro8 showed maximum zone of inhibition, 12.5 and 16.8 mm, respectively. Minimum inhibitory zones observed in *E. faecium* SBBPro7 and *B. subtilis* SBBPro9 were 11.5 and 12.8 mm against amoxil and ciprofloxacin, respectively. In case of azithromycin and erythromycin, *L. rhamnosus* SBBPro6 showed maximum zones of inhibition, 11.33 and 11 mm, respectively, while minimum inhibitory zones were exhibited by *B. amyloliquefaciens* SBBPro8 (7.5 mm) and *E. faecium* SBBPro7 (6 mm) (Supplementary Table S12, Figure 9). All the probiotics demonstrated statistically significant sensitivity against all the antibiotics at all three concentrations with *p*-values < 0.005 in each case (Supplementary Tables S13a–e).

## 4 Discussion

The five isolates characterized in this study were identified as *L. rhamnosus, E. faecium, B. amyloliquefaciens, B. subtilis*, and *B. cereus. Lactobacillus*, which has been granted Generally Recognized as Safe (GRAS) status, is of particular interest due to its indigenous origin from the gut, enhancing its potential application as a probiotic in aquaculture. Several studies have previously documented *L. rhamnosus* (78), *E. faecium* (79–82), *B. amyloliquefaciens* (83, 84), *B. subtilis* (85–87), and *B. cereus* (88) as fish gut-associated bacteria, supporting the findings of this study. Additionally, the Gram-positive nature of the identified isolates is consistent with current literature (89, 90). Earlier explored GIT-associated probiotics from marine fishes documented so far include genera of *Lactobacillus, Bacillus*, and *Enterococcus* (69, 91, 92). Hence, this study is in accordance with the previously published study.

A study has documented fermentation potentials for different carbohydrates in gut-borne lactic acid bacterial strains in marine fishes, such as mannitol (strains LB41 and LC1333) and glucose and fructose (strains LB411, LE823, LC1132, LC1333, LC1334, LC1342, and LC1344) (69). These findings are consistent with the current study's results.

Tolerance to bile salts and an acidic environment are two of the major prerequisites for bacteria to survive in the fish gut during transit through the stomach and intestine. The isolates from this study demonstrated growth at pH values of 3 and 5 and at a bile concentration of 0.3 g. This suggests that these bacteria do have the potential to survive well in the fish gut environment. Consistent with our finding, *L. plantarum* LB411 from the marine fish gut has been documented to exhibit optimal growth at pH = 3. In contrast, *Enterococcus faecium* EN936 *and E. gallinarum* EN10113 have been reported to grow only at near-neutral pH (69). The response of the bacterial isolates to varying pH levels aligns with previous literature, as most gut microbes have been documented to exhibit more tolerance to pH > 3 (89). Additionally, all isolates in our study exhibited significant growth at the exponential phase, ranging between 1.91 ± 0.02 and 2.10 ± 0.07 at 0.3% bile salt concentration. However, this finding does not appear to be consistent with previous literature (89). Optimal bile salt tolerance in marine fish gut bacteria is reported as 1% in *L. mesenteroides* LE823, *L. plantarum* LB411, and *E. gallinarum* EN10113, while the least tolerance is reported at 0.1% concentration in *Lactococci* strains (69).

All isolates from this study exhibited the highest tolerance at 0.2 (OD^600^ of 1.61 to 1.86) and 2% (OD^600^ of 1.45 to 1.62) NaCl concentration. In contrast, previous studies have reported optimal bacterial growth in fish gut isolates at 0.1% (OD^600^ of 0.25 to 0.1) and markedly reduced growth (OD^600^ of 0.025 to 0.125) at 2% salt concentration (90). Hence, the findings of this study contradict those previously reported.

All isolates demonstrated tolerance to synthetic gastric fluid, and this indicates their ability to withstand gastrointestinal conditions in the host, further confirming their safety profile as probiotics. This finding is also consistent with earlier reported literature (66, 93).

In the current study, isolates exhibited optimum growth at 2 and 3 pH. However, as the pH approached neutrality (pH 5), growth decreased, and this finding contradicts previous literature reporting optimal growth at near-neutral pH values (5 and 9). A previous study also documented optimal growth at bile salt concentrations of 0.5% and 1%, which is partially consistent with this study, where isolates showed tolerance at 0.3% bile salt concentration (69). Additionally, another study reported the optimal growth of fish gut probiotics at neutral pH values (pH 6 and 7) (90), whereas a separate study documented the highest survival rates at pH 2 and 3 with 2% bile salt concentration (34). These findings align with the current study (94).

The ⋎ hemolysis exhibited by the isolates from this study is consistent with previously published research (34, 95, 96). A study on marine fish gut-borne bacteria also showed no hemolysis (97). However, contrary to these findings, some bacterial isolates have been reported to exhibit beta hemolysis (98).

Several studies have evaluated the antimicrobial potential of fish gut bacteria against pathogens that were identified in this study (34). One study classified these bacteria among major fish food-associated pathogens and further documented the antimicrobial activity of fish gut bacteria against *P. aeruginosa* and *S. aureus* (99). While the majority of bacteria were insensitive to *S. aureus* and *P. aeruginosa*, the findings of the current study are consistent with previous research documenting fish gut probiotics with resistance to *S. aureus* (100–102). Consistent with current findings, another study has reported antagonistic activity in marine fish gut-borne bacteria against *P. aeruginosa* and *S. aureus* (97).

The isolates from this study exhibited resistance to heat shock at 100°C. However, previous literature has reported fish-borne bacteria tolerating temperatures only up to 30°C and 37°C (90). Consistent with the present findings, other studies have reported fish-borne bacteria tolerating heat shocks at 80°C, 90°C, and 100°C (96).

Probiotic bacteria with strong potential must be capable of reducing cholesterol levels to help maintain a healthy threshold in the host (103). In this study, the isolates *B. cereus, B. subtilis*, and *B. amyloliquefaciens* exhibited cholesterol assimilation potentials of 34 mg/dL, 39 mg/dL, and 70 mg/dL, respectively. These findings are supported by previous studies that reported cholesterol metabolism roles of these species in Atlantic salmon, Amur minnow, and rohu (104–106). The isolates from this study exhibited cholesterol assimilation within a range of 26.76 to 83.44 mg/dL, which contradicts another study that documented cholesterol assimilation efficiencies between 1.2 and 4.3 mg/dL in fish gut probiotics (107).

The transfer of ARGs remains a major safety concern in using probiotics as feed supplements (108). However, all isolated strains in this study were highly sensitive to antibiotics, as demonstrated by the clear inhibition zones observed. Sensitivity to erythromycin was particularly consistent with existing literature (89, 109). Furthermore, various studies have reported antibiotic sensitivity in freshwater as well as marine fish gut bacterial species (34, 97). The use of these bacterial isolates in aquaculture could prevent the dispersion and enrichment of ARGs within aquatic systems.

Literature data regarding the gut bacteria of *S. solea* is limited. However, several articles have reported the effect of probiotic supplementation on larviculture improvement of *S. solea* (110) and juvenile intestine function and growth, immune response, gut morphology, host defense, ecology of digestive tract, and gut microbial diversity in *Solea senegalensis* (111–114). To the best of our knowledge, only two studies so far have reported the isolation and characterization of *S. solea* gut-borne bacteria internationally. On the other hand, in Pakistan, this is the first ever study reporting gut microbes of this marine fish (115, 116). Hence, this study documents the information regarding the probiotic potential of gut microbes from a least-explored fish.

## 5 Conclusion

The isolates characterized in this study demonstrated strong probiotic potential based on their excellent *in vitro* characterization. Antibiotic sensitivity of these isolates is a significant finding of the current project and indicates the potential of documented bacteria as a safe alternative to antibiotics. Additionally, the results from the simulated gastric medium survival assay and optimal growth at pH 2 and 3 suggested that these isolates could adapt to the gastric conditions in the host. Keeping in view these probiotic attributes, bacterial strains might be recommended for *in vivo* assessment of their impact on fish growth, body weight, and meat quality through their in-feed administration.

## Data Availability

The FASTA sequences of present study probiotics are available at NCBI database. The accession numbers assigned are PQ002180, PQ002492, PQ002184, PQ002187, and PQ002188.
