# Supplementary material for: In vitro analysis of probiotic characteristics of gut-associated bacteria from Solea solea
Source: Front Vet Sci. 2025 Jun 4;12:1581675. doi: 10.3389/fvets.2025.1581675 (PMC12175249; doi:10.3389/fvets.2025.1581675)
Supplement: Supplementary file 1 [file Data_Sheet_1.docx]

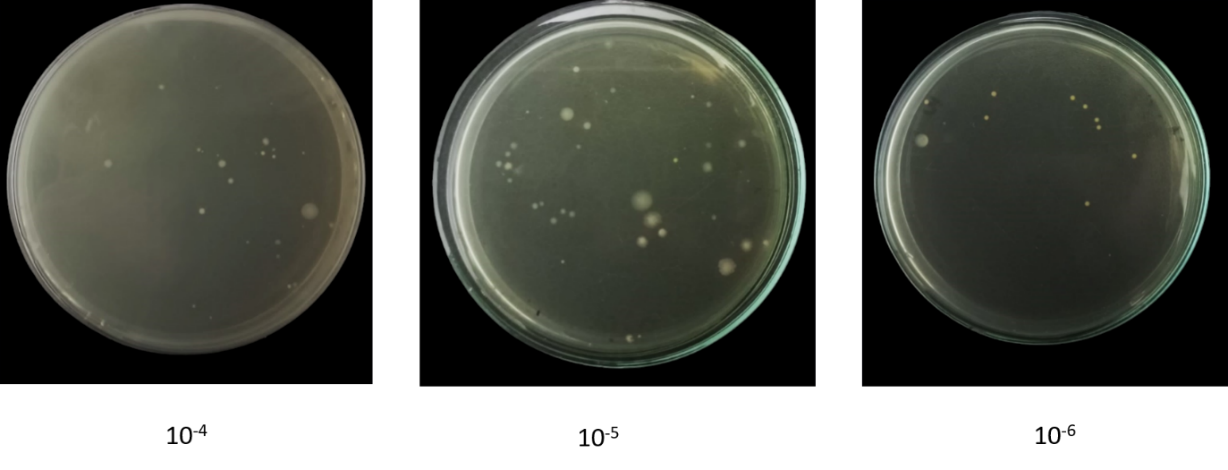


Supplementary Data Figure S1: Colony forming units (CFUs) of bacteria documented in current study obtained through serial dilution method


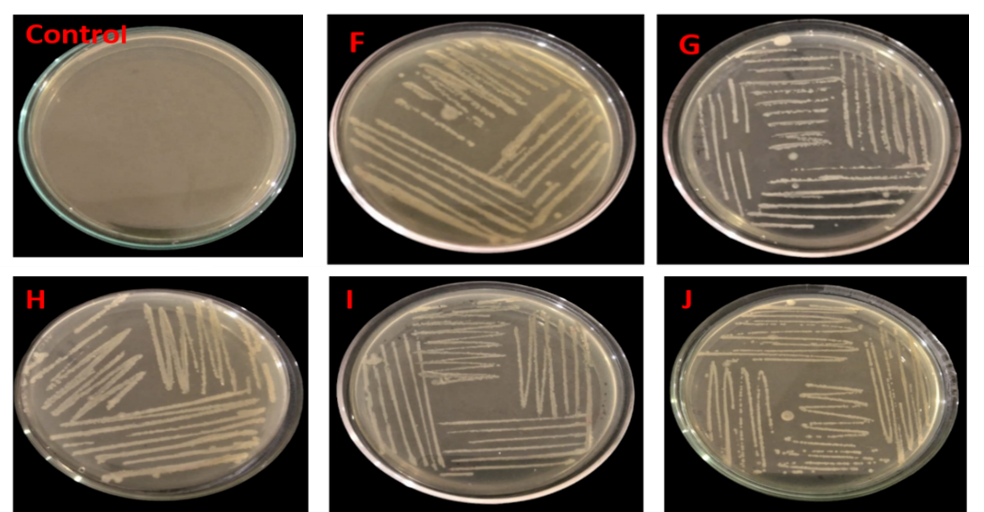


**Supplementary Data Figure S2**: Morphological characterization of bacteria isolated in current study through analysis of texture, shape, color, margins and elevations of colonies

F) *L. rhamnosus* SBBPro6 G) *E. faecium* SBBPro7 H) *B. amyloliquefaciens* SBBPro8 I) *B. subtilis* SBBPro9 J) *B. cereus* SBBPro10


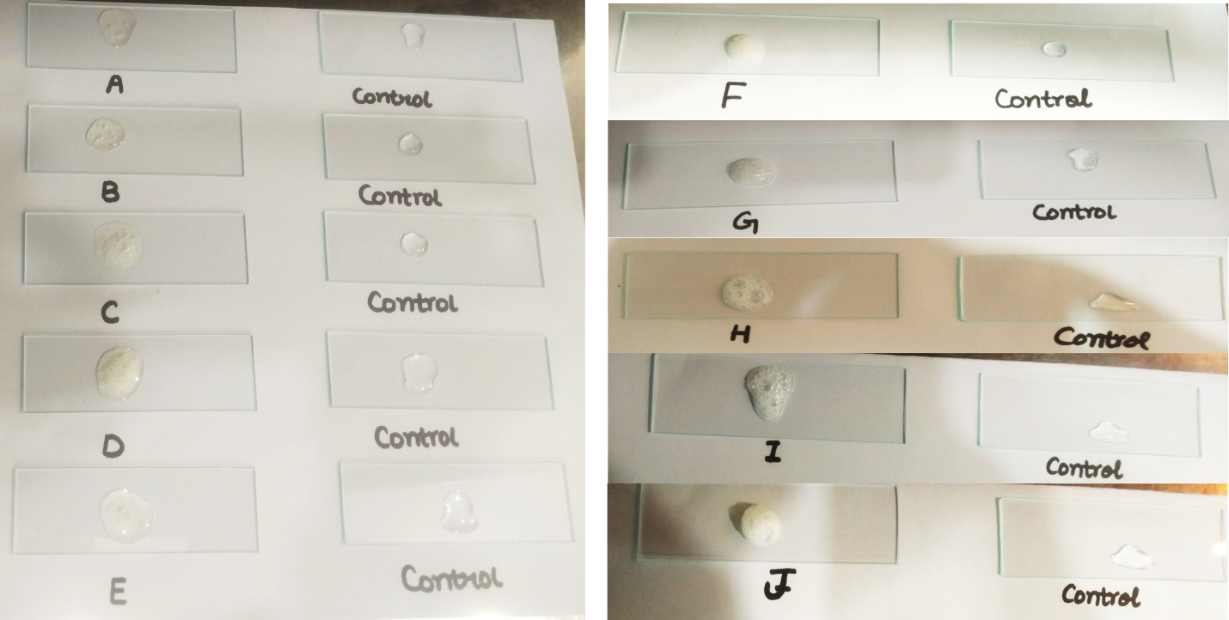


**Supplementary Data Figure S3**: Biochemical characterization of bacteria isolated in current study by catalase test

F) *L. rhamnosus* SBBPro6 G) *E. faecium* SBBPro7 H) *B.* *amyloliquefaciens* SBBPro8 I) *B****.*** *subtilis* SBBPro9 and J) *B.* *cer****e****us* SBBPro10

**
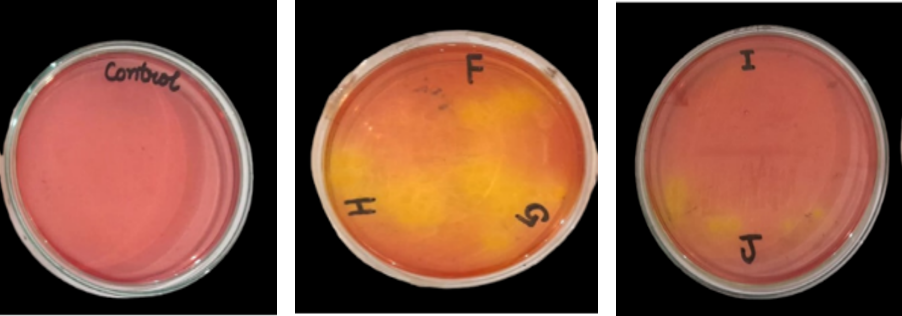
**

Supplementary Data Figure S4: Biochemical characterization of bacteria isolated in current study through mannitol salt agar (MSA) test

F) L. rhamnosus SBBPro6 G) E. faecium SBBPro7 H) B. amyloliquefaciens SBBPro8 I) B. subtilis SBBPro9 and J) B. cereus SBBPro10


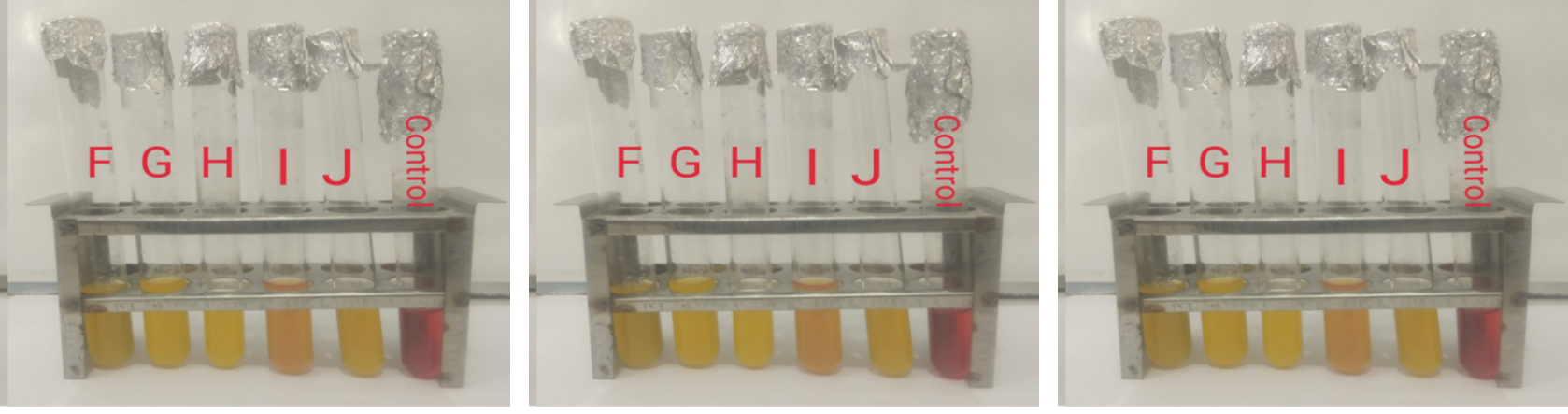


Supplementary Data Figure S5: Biochemical characterization of bacteria isolated in current study through glucose, lactose and fructose fermentation tests

F) L. rhamnosus SBBPro6 G) E. faecium SBBPro7 H) B. amyloliquefaciens SBBPro8 I) B. subtilis SBBPro9 and J) B. cereus SBBPro10


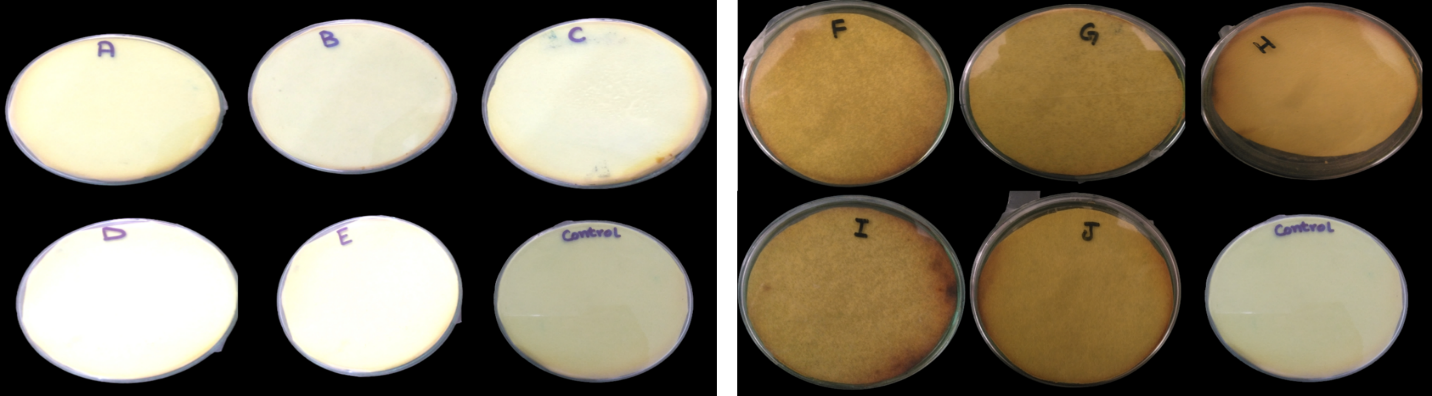


Supplementary Data Figure S6: Biochemical characterization of bacteria isolated in current study through HCN production test

F) L. rhamnosus SBBPro6 G) E. faecium SBBPro7 H) B. amyloliquefaciens SBBPro8 I) B. subtilis SBBPro9 and J) B. cereus SBBPro10


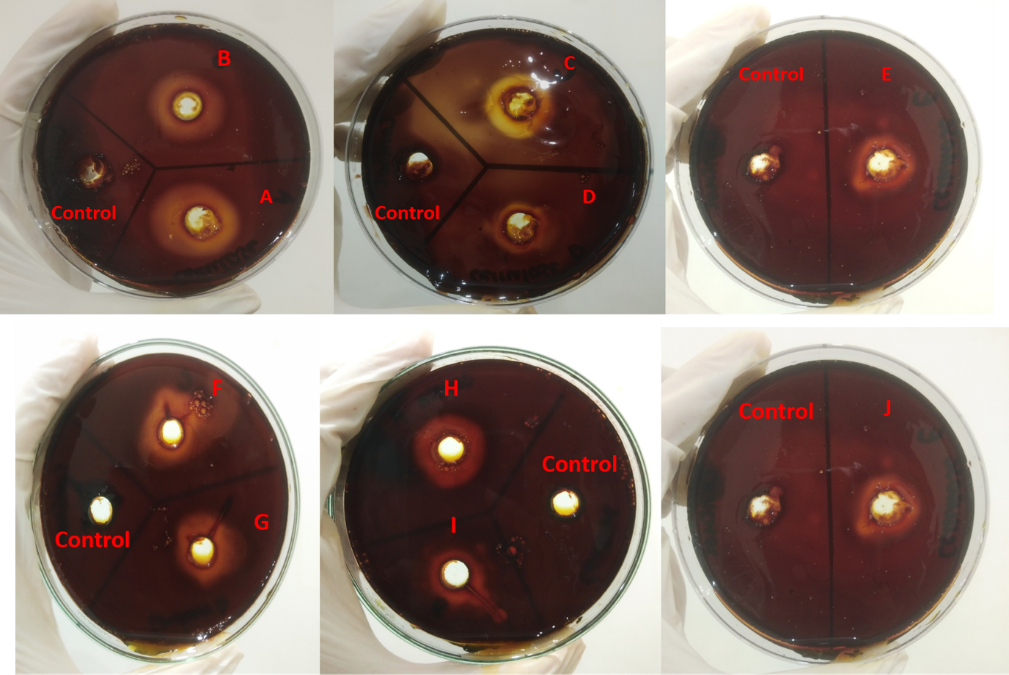


**Supplementary Data Figure S7:** Biochemical characterization of bacteria isolated in current study through cellulase production test

F) L. rhamnosus SBBPro6 G) E. faecium SBBPro7 H) B. amyloliquefaciens SBBPro8 I) B. subtilis SBBPro9 and J) B.cereus SBBPro10


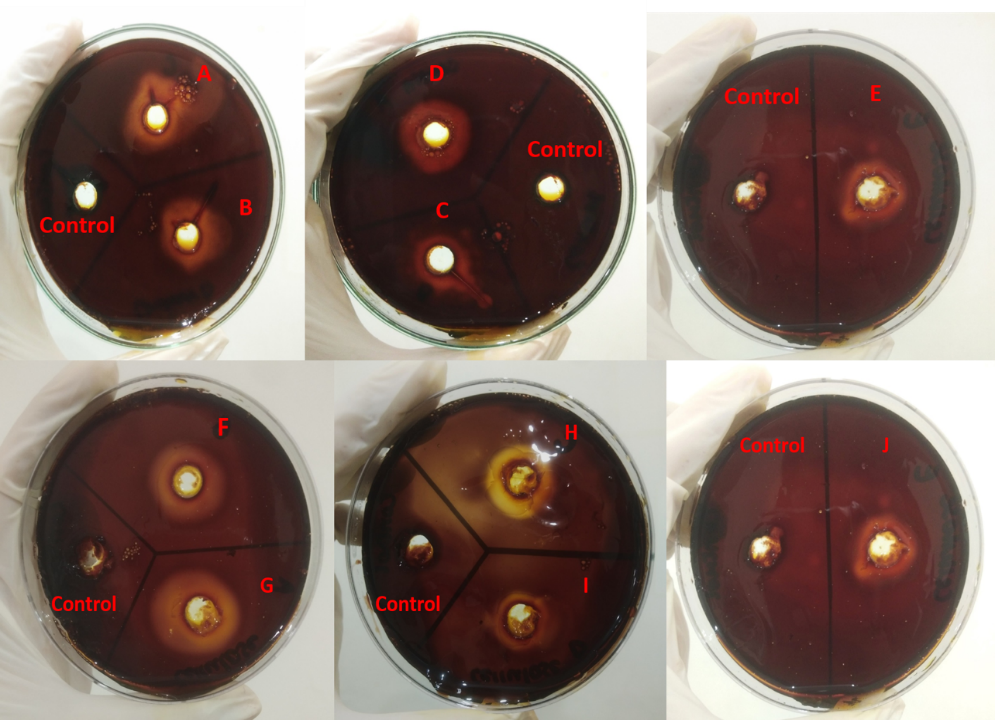


**Supplementary Data Figure S8**: Biochemical characterization of bacteria isolated in current study through chitinase production test

F) L. rhamnosus SBBPro6 G) E. faecium SBBPro7 H) B. amyloliquefaciens SBBPro8 I) B. subtilis SBBPro9 and J) B.cereus SBBPro10


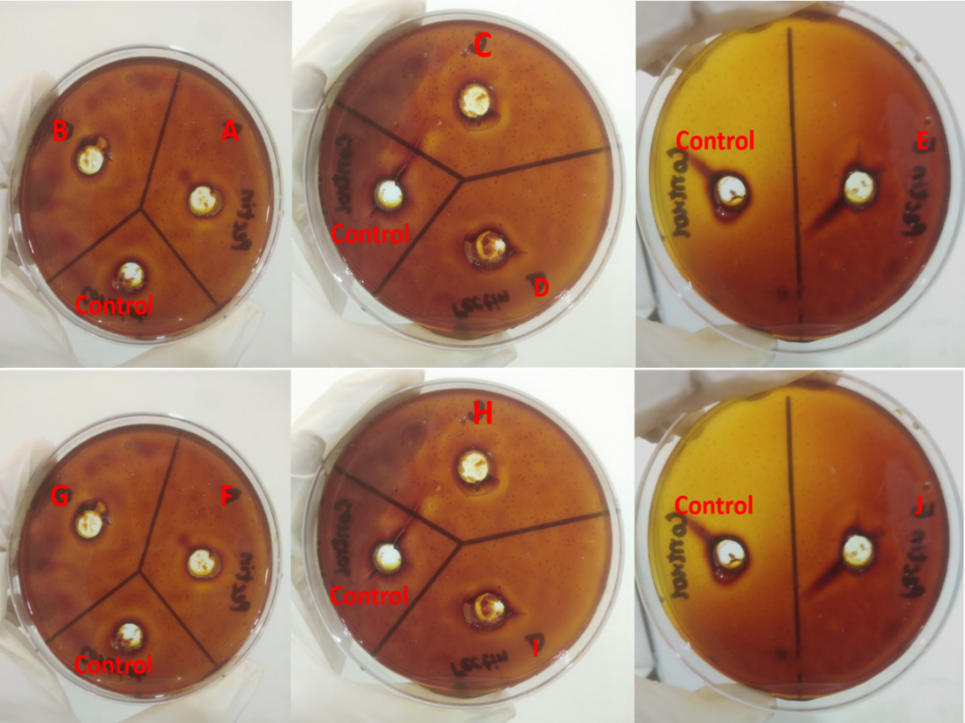


**Supplementary Data Figure S9:** Biochemical characterization of bacteria isolated in current study through pectinase production test

F) L. rhamnosus SBBPro6 G) E. faecium SBBPro7 H) B. amyloliquefaciens SBBPro8 I) B. subtilis SBBPro9 and J) B.cereus SBBPro10


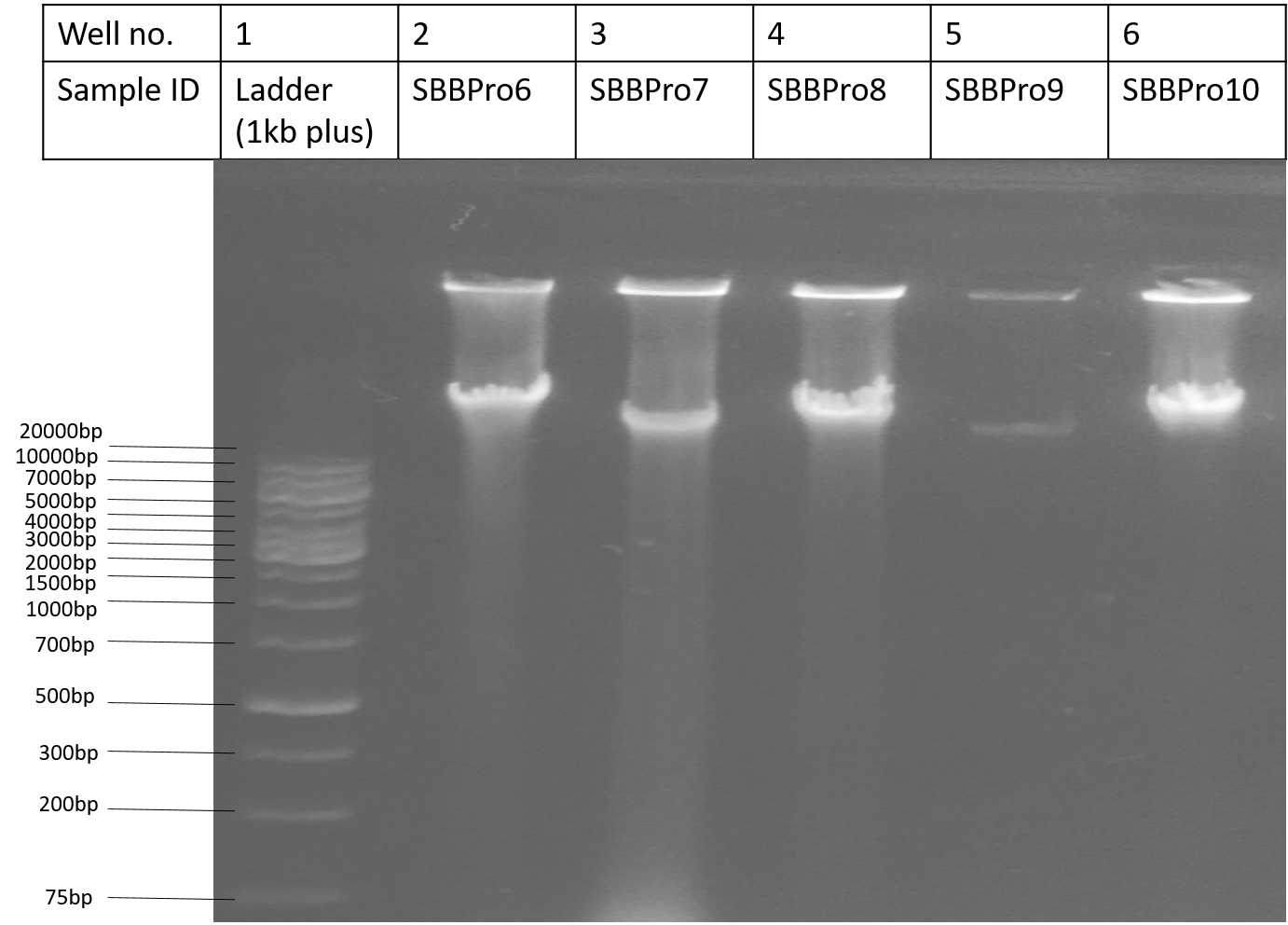


**Supplementary Data Figure S10:** Visualization of DNA samples extracted from current study documented bacteria through agarose gel electrophoresis

**
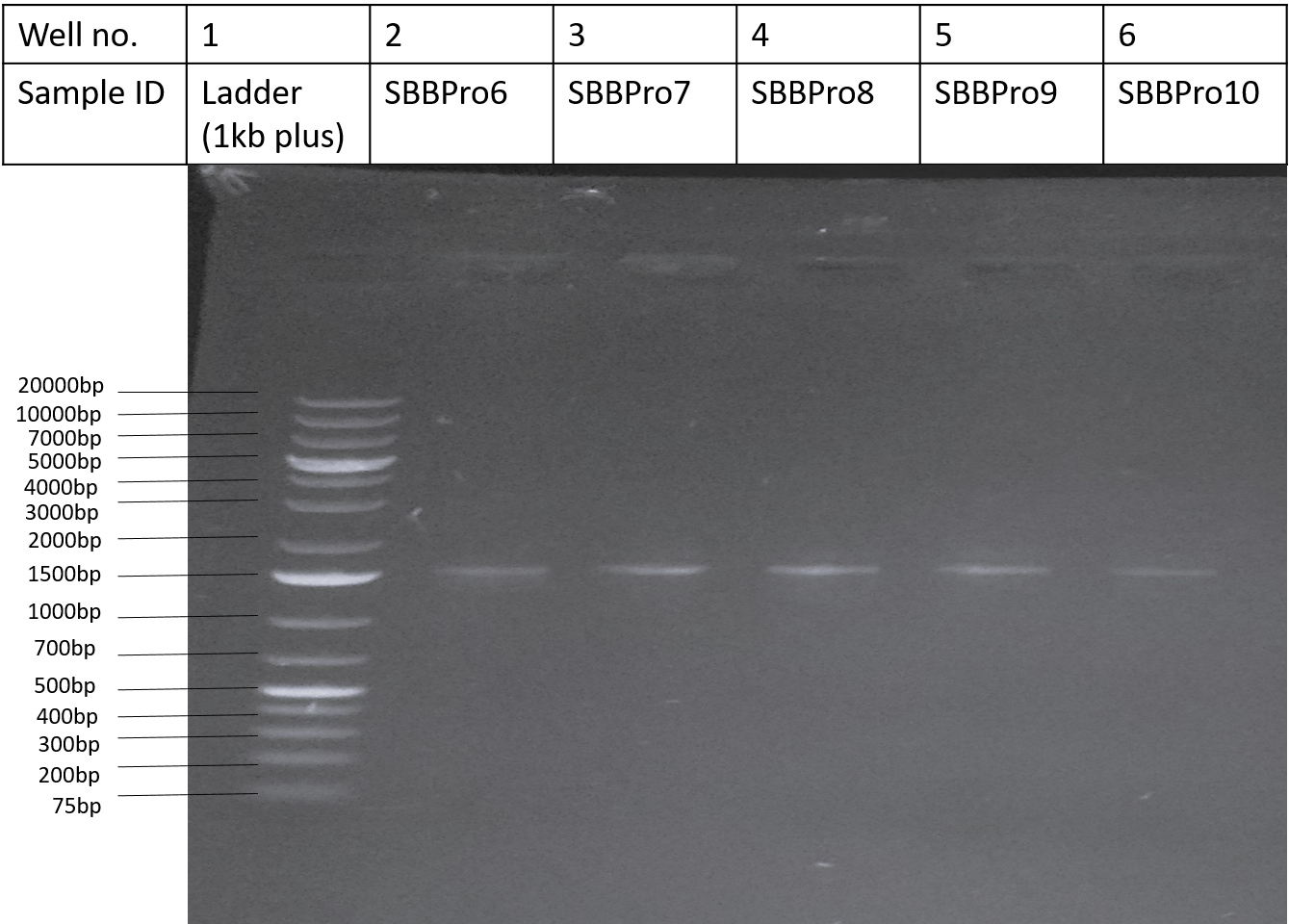
**

**Supplementary Data Figure S11:** PCR amplicons of current study documented bacteria resolved on agarose gel

**
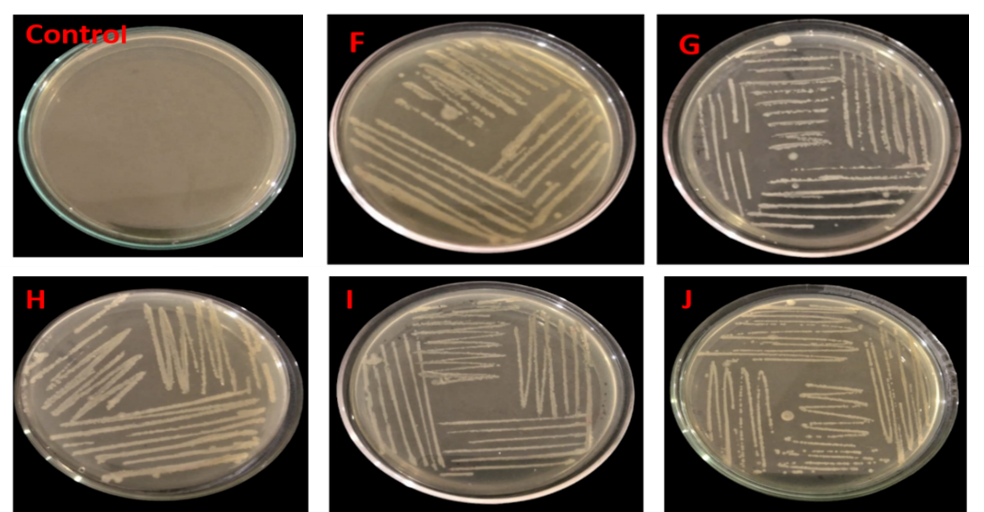
**

Supplementary Data Figure S12: Assessment of survival potential of current study documented bacteria under simulated gastric conditions

F) L. rhamnosus SBBPro6 G) E. faecium SBBPro7 H) B. amyloliquefaciens SBBPro8 I) B. subtilis SBBPro9 and J) B.cereus SBBPro10

**
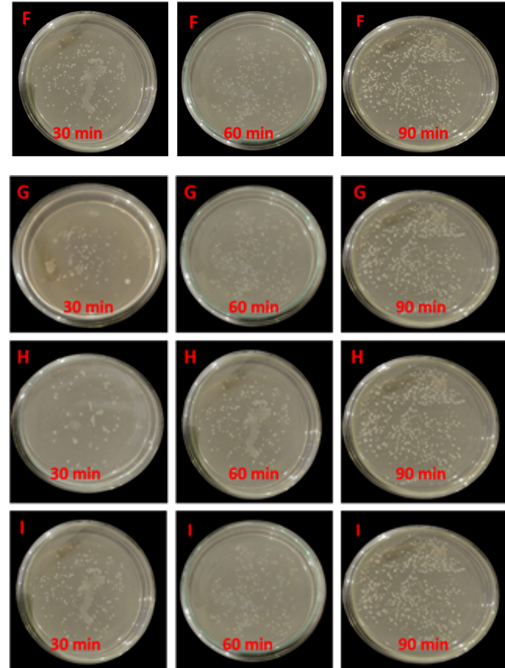
**

**Supplementary Data Figure S13:** Assessment of adhesion potential of fish gut borne bacteria with intestinal cells at three different time intervals

F) L. rhamnosus SBBPro6 G) E. faecium SBBPro7 H) B. amyloliquefaciens SBBPro8 I) B. subtilis SBBPro9 and J) B.cereus SBBPro10

**
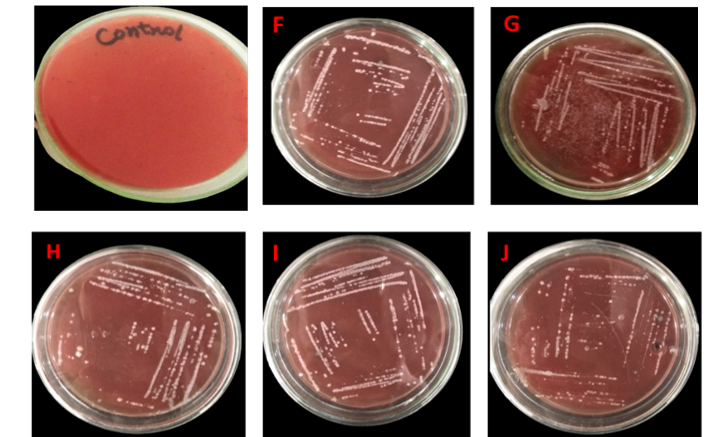
**

**Supplementary Data Figure S14:** Assessment of hemolytic potential of bacteria isolated in current study via hemolytic assay

F) L. rhamnosus SBBPro6 G) E. faecium SBBPro7 H) B. amyloliquefaciens SBBPro8 I) B. subtilis SBBPro9 and J) B. cereus SBBPro10

**
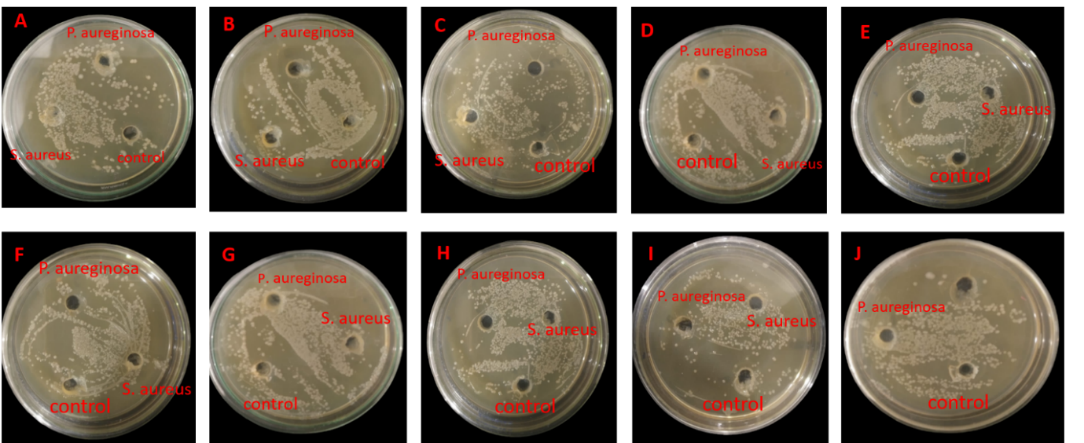
**

Supplementary Data Figure S15: Assessment of antimicrobial resistance potential of current study documented bacteria against Pseudomonas aeruginosa and Staphylococcus aureus

F) L. rhamnosus SBBPro6 G) E. faecium SBBPro7 H) B. amyloliquefaciens SBBPro8 I) B. subtilis SBBPro9 and J) B. cereus SBBPro10


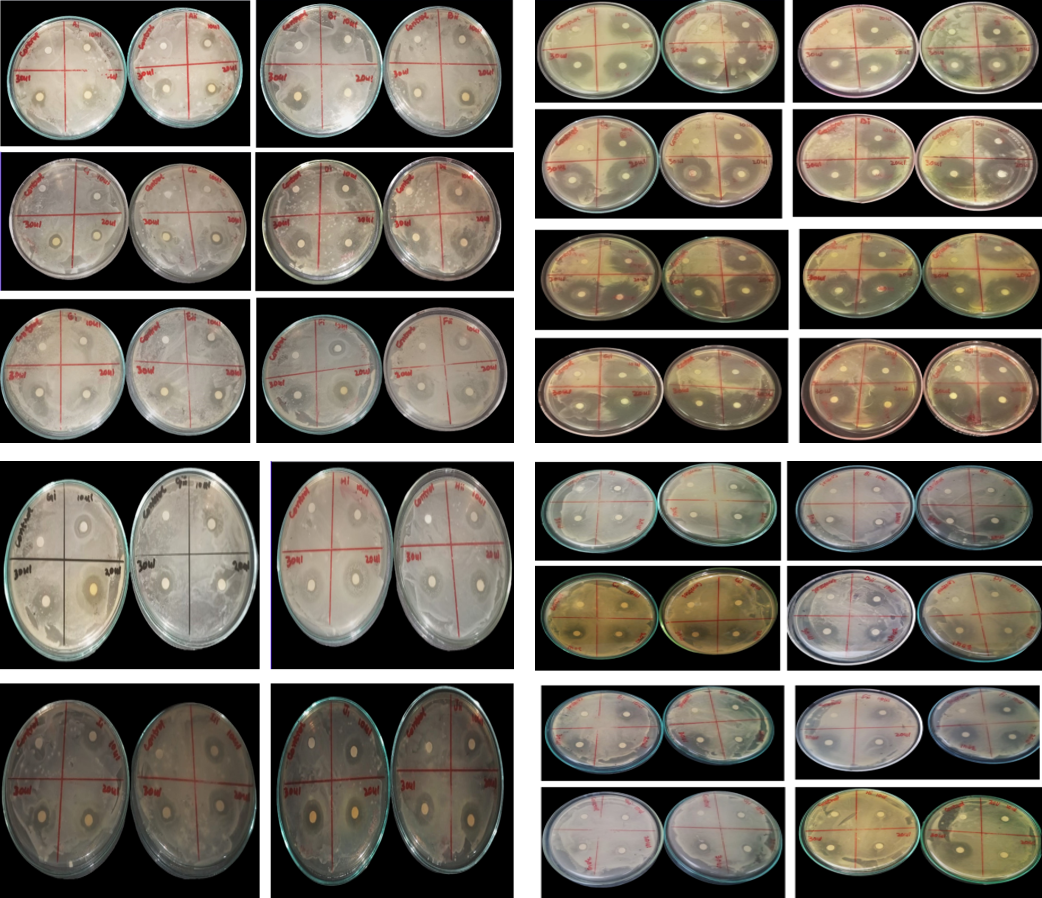


Supplementary Data Figure S16: Antibiotic sensitivity profiling of bacteria isolated in current study against antibiotics amoxil, azithromycin, erythromycin, velosef and ciprofloxacin

F) L. rhamnosus SBBPro6 G) E. faecium SBBPro7 H) B. amyloliquefaciens SBBPro8 I) B. subtilis SBBPro9 and J) B. cereus SBBPro10

Supplementary Data Table S1: Biochemical characterization of current study documented bacteria

| **Isolates** | **Cat** | **MNL** | **GLU** | **LAC** | **FRU** | **HCN** | **Cell** | **Chi** | **Pec** |
| --- | --- | --- | --- | --- | --- | --- | --- | --- | --- |
| *Lacticaseibacillus rhamnosus* SBBPro6 | + | + | + | + | + | + | + | + | - |
| *Enterococcus faecium* SBBPro7 | + | + | + | + | + | + | + | + | - |
| *Bacillus amyloliquefaciens* SBBPro8 | + | + | + | + | + | + | + | + | - |
| *Bacillus subtilis* SBBPro9 | + | - | + | + | + | + | + | + | - |
| *Bacillus cereus* SBBPro10 | + | + | + | + | + | + | + | + | - |

Cat: catalase production test, MNL: mannitol fermentation test, GLU: glucose fermentation test, LAC: lactose fermentation test, FRU: fructose fermentation test, HCN: HCN production test, Cell: cellulase production test, Chi: chitinase production test, Pec: pectinase production test

Supplementary Data Table S2: Analysis of growth phases of current study documented bacteria through measurement of OD^600^ at different time intervals

| **Isolates** | **OD^600^ at different intervals (hours)** | | | | | | | | |
| --- | --- | --- | --- | --- | --- | --- | --- | --- | --- |
|  | **0** | **3** | **6** | **24** | **27** | **30** | **48** | **51** | **54** |
| *Lacticaseibacillus rhamnosus* SBBPro6 | 0.57± 0.024 | 0.961± 0.031 | 1.026± 0.017 | 1.042± 0.015 | 1.127± 0.024 | 1.116± 0.038 | 1.099± 0.043 | 1.028± 0.030 | 1.011± 0.046 |
| *Enterococcus faecium* SBBPro7 | 0.515± 0.008 | 0.945± 0.013 | 0.978± 0.023 | 1.04± 0.04 | 1.490± 0.003 | 1.394± 0.014 | 1.333± 0.040 | 1.065± 0.005 | 1.055± 0.001 |
| *Bacillus amyloliquefaciens* SBBPro8 | 0.601± 0.001 | 0.957± 0.017 | 0.965± 0.013 | 1.013± 0.009 | 1.033± 0.007 | 1.172± 0.01 | 1.121± 0.016 | 1.055± 0.008 | 0.188± 0.009 |
| *Bacillus subtilis* SBBPro9 | 0.663± 0.012 | 0.808± 0.005 | 1.006± 0.001 | 1.108± 0.049 | 1.117± 0.008 | 1.356± 0.044 | 1.220± 0.052 | 1.205± 0.001 | 0.227± 0.002 |
| *Bacillus cereus* SBBPro10 | 0.636± 0.010 | 0.808± 0.017 | 1.014± 0.001 | 1.024± 0.0001 | 1.081± 0.002 | 1.146± 0.007 | 1.098± 0.014 | 1.049± 0.046 | 0.111± 0.002 |

Supplementary Data Table S3: Assessment of bile salt tolerance of current study documented bacteria at 0.3% concentration based on measurement of OD^600^ at log phase

| **Isolates** | **OD^600^ at log phase** | **p-value** |
| --- | --- | --- |
| *Lacticaseibacillus rhamnosus* SBBPro6 | 1.95 ± 0.05 | 0.05 |
| *Enterococcus faecium* SBBPro7 | 1.91 ± 0.02 |  |
| *Bacillus amyloliquefaciens* SBBPro8 | 2.09 ± 0.16 |  |
| *Bacillus subtilis* SBBPro9 | 1.96 ± 0.07 |  |
| *Bacillus cereus* SBBPro10 | 2.10 ± 0.07 |  |

**Supplementary Data Table S4a:** Statistical analysis of bile salt tolerance test of current study documented bacteria through One-Way Anova

|  | Sum of Squares | df | Mean Square | F | Significance |
| --- | --- | --- | --- | --- | --- |
| Between Groups | 3.567 | 5 | .713 | 116.838 | .000 |
| Within Groups | .085 | 14 | .006 |  |  |
| Total | 3.653 | 19 |  |  |  |

**Supplementary Data Table S4b:** Statistical analysis of bile salt tolerance assay of current study documented bacteria through Dunnett t-test

| (I) Probiotics | | Mean Difference (I-J) | Std. Error | Sig. | 95% Confidence Interval | |
| --- | --- | --- | --- | --- | --- | --- |
|  |  |  |  |  | Lower Bound | Upper Bound |
| *Lacticaseibacillus rhamnosus* SBBPro6 | Control | .91160^*^ | .05707 | .000 | .7471 | 1.0761 |
| *Enterococcus faecium* SBBPro7 | Control | .86760^*^ | .05707 | .000 | .7031 | 1.0321 |
| *Bacillus amyloliquefaciens* SBBPro8 | Control | 1.05393^*^ | .05707 | .000 | .8894 | 1.2185 |
| *Bacillus subtilis* SBBPro9 | Control | .92293^*^ | .05707 | .000 | .7584 | 1.0875 |
| *Bacillus cereus* SBBPro10 | Control | 1.05760^*^ | .05707 | .000 | .8931 | 1.2221 |

**Supplementary Data Table S5:** Assessment of NaCl tolerance potential of current study documented bacteria based on measurement of OD^600^ at log phase at different concentrations of NaCl

| **Isolates** | **OD^600^ at log phase** | | |  |
| --- | --- | --- | --- | --- |
|  | **0.2g** | **2g** | **5g** | **p-value** |
| *Lacticaseibacillus rhamnosus* SBBPro6 | 1.86 ± 0.04 | 1.62 ± 0.15 | 1.44 ± 0.01 | 0.05 |
| *Enterococcus faecium* SBBPro7 | 1.76 ± 0.06 | 1.47 ± 0.06 | 1.15 ± 0.04 |  |
| *Bacillus amyloliquefaciens* SBBPro8 | 1.78 ± 0.03 | 1.58 ± 0.05 | 1.19 ± 0.01 |  |
| *Bacillus subtilis* SBBPro9 | 1.65 ± 0.04 | 1.45 ± 0.03 | 1.16 ± 0.10 |  |
| *Bacillus cereus* SBBPro10 | 1.61 ± 0.11 | 1.50 ± 0.04 | 1.33 ± 0.07 |  |

**Supplementary Data Table S6a:** Statistical analysis of NaCl tolerance assay of current study documented bacteria at 0.2g concentration through One-Way Anova

|  | Sum of Squares | df | Mean Square | F | Sig. |
| --- | --- | --- | --- | --- | --- |
| Between Groups | 1.902 | 5 | .380 | 104.873 | .000 |
| Within Groups | .051 | 14 | .004 |  |  |
| Total | 1.953 | 19 |  |  |  |

**Supplementary Data Table S6b:** Statistical analysis of NaCl tolerance assay of current study documented bacteria at 0.2g concentration through Dunnett t-test

| (I) Probiotics | | Mean Difference (I-J) | Std. Error | Sig. | 95% Confidence Interval | |
| --- | --- | --- | --- | --- | --- | --- |
|  |  |  |  |  | Lower Bound | Upper Bound |
| *Lacticaseibacillus rhamnosus* SBBPro6 | Control | .81993^*^ | .04399 | .000 | .6931 | .9467 |
| *Enterococcus faecium* SBBPro7 | Control | .71527^*^ | .04399 | .000 | .5885 | .8421 |
| *Bacillus amyloliquefaciens* SBBPro8 | Control | .73460^*^ | .04399 | .000 | .6078 | .8614 |
| *Bacillus subtilis* SBBPro9 | Control | .60460^*^ | .04399 | .000 | .4778 | .7314 |
| *Bacillus cereus* SBBPro10 | Control | .56693^*^ | .04399 | .000 | .4401 | .6937 |

**Supplementary Data Table S6c:** Statistical analysis of NaCl tolerance assay of current study documented bacteria at 2g concentration through One-Way Anova

|  | Sum of Squares | df | Mean Square | F | Sig. |
| --- | --- | --- | --- | --- | --- |
| Between Groups | .886 | 5 | .177 | 32.821 | .000 |
| Within Groups | .076 | 14 | .005 |  |  |
| Total | .961 | 19 |  |  |  |

**Supplementary Data Table S6d:** Statistical analysis of NaCl tolerance assay of current study documented bacteria at 2g concentration through Dunnett t-test

| (I) Probiotics | | Mean Difference (I-J) | Std. Error | Sig. | 95% Confidence Interval | |
| --- | --- | --- | --- | --- | --- | --- |
|  |  |  |  |  | Lower Bound | Upper Bound |
| *Lacticaseibacillus rhamnosus* SBBPro6 | Control | .57660^*^ | .05365 | .000 | .4219 | .7313 |
| *Enterococcus faecium* SBBPro7 | Control | .43193^*^ | .05365 | .000 | .2773 | .5866 |
| *Bacillus amyloliquefaciens* SBBPro8 | Control | .48560^*^ | .05365 | .000 | .3309 | .6403 |
| *Bacillus subtilis* SBBPro9 | Control | .40860^*^ | .05365 | .000 | .2539 | .5633 |
| *Bacillus cereus* SBBPro10 | Control | .45627^*^ | .05365 | .000 | .3016 | .6109 |

**Supplementary Data Table S7:** Assessment of pH tolerance potential of current study documented bacteria at pH values 2, 3 and 5 through measurement of OD^600^

| **Isolates** | **OD^600^ at pH 2** | **p-value** | **OD^600^ at pH 3** | **p-value** | **OD^600^ at pH 5** | **p-value** |
| --- | --- | --- | --- | --- | --- | --- |
| *Lacticaseibacillus rhamnosus* SBBPro6 | 0.40 ± 0.006 | 0.05 | 0.70 ± 0.002 | 0.05 | 0.94 ± 0.007 | 0.05 |
| *Enterococcus faecium* SBBPro7 | 0.44 ± 0.042 |  | 0.79 ± 0.001 |  | 0.85 ± 0.044 |  |
| *Bacillus amyloliquefaciens* SBBPro8 | 0.48 ± 0.009 |  | 0.72 ± 0.026 |  | 1.07 ± 0.082 |  |
| *Bacillus subtilis* SBBPro9 | 0.43 ± 0.035 |  | 0.77 ± 0.009 |  | 0.93 ± 0.007 |  |
| *Bacillus cereus* SBBPro10 | 0.29 ± 0.080 |  | 0.84 ± 0.019 |  | 1.04 ± 0.057 |  |

**Supplementary Data Table S8a:** Statistical analysis of pH tolerance assay (pH = 2) of current study documented bacteria through One-Way Anova

|  | Sum of Squares | df | Mean Square | F | Sig. |
| --- | --- | --- | --- | --- | --- |
| Between Groups | 1.564 | 5 | .313 | 177.736 | .000 |
| Within Groups | .025 | 14 | .002 |  |  |
| Total | 1.589 | 19 |  |  |  |

**Supplementary Data Table S8b:** Statistical analysis of pH tolerance assay (pH = 2) of current study documented bacteria through Dunnett t-test

| (I) Probiotics | | Mean Difference (I-J) | Std. Error | Sig. | 95% Confidence Interval | |
| --- | --- | --- | --- | --- | --- | --- |
|  |  |  |  |  | Lower Bound | Upper Bound |
| *Lacticaseibacillus rhamnosus* SBBPro6 | Control | -.64407^*^ | .03064 | .000 | -.7324 | -.5557 |
| *Enterococcus faecium* SBBPro7 | Control | -.59740^*^ | .03064 | .000 | -.6857 | -.5091 |
| *Bacillus amyloliquefaciens* SBBPro8 | Control | -.56040^*^ | .03064 | .000 | -.6487 | -.4721 |
| *Bacillus subtilis* SBBPro9 | Control | -.60673^*^ | .03064 | .000 | -.6951 | -.5184 |
| *Bacillus cereus* SBBPro10 | Control | -.75273^*^ | .03064 | .000 | -.8411 | -.6644 |

**Supplementary Data Table S8c:** Statistical analysis of pH tolerance assay (pH = 3) of current study documented bacteria through One-Way Anova

|  | Sum of Squares | df | Mean Square | F | Sig. |
| --- | --- | --- | --- | --- | --- |
| Between Groups | .329 | 5 | .066 | 117.783 | .000 |
| Within Groups | .008 | 14 | .001 |  |  |
| Total | .337 | 19 |  |  |  |

**Supplementary Data Table S8d:** Statistical analysis of pH tolerance assay (pH = 3) of current study documented bacteria through Dunnett t-test

| (I) Probiotics | | Mean Difference (I-J) | Std. Error | Sig. | 95% Confidence Interval | |
| --- | --- | --- | --- | --- | --- | --- |
|  |  |  |  |  | Lower Bound | Upper Bound |
| *Lacticaseibacillus rhamnosus* SBBPro6 | Control | -.34407^*^ | .01726 | .000 | -.3938 | -.2943 |
| *Enterococcus faecium* SBBPro7 | Control | -.24607^*^ | .01726 | .000 | -.2958 | -.1963 |
| *Bacillus amyloliquefaciens* SBBPro8 | Control | -.32407^*^ | .01726 | .000 | -.3738 | -.2743 |
| *Bacillus subtilis* SBBPro9 | Control | -.27207^*^ | .01726 | .000 | -.3218 | -.2223 |
| *Bacillus cereus* SBBPro10 | Control | -.20240^*^ | .01726 | .000 | -.2522 | -.1526 |

**Supplementary Data Table S8e:** Statistical analysis of pH tolerance assay (pH = 5) of current study documented bacteria through One-Way Anova

|  | Sum of Squares | df | Mean Square | F | Sig. |
| --- | --- | --- | --- | --- | --- |
| Between Groups | .118 | 5 | .024 | 11.066 | .000 |
| Within Groups | .030 | 14 | .002 |  |  |
| Total | .148 | 19 |  |  |  |

**Supplementary Data Table S8f:** Statistical analysis of pH tolerance assay (pH = 5) of current study documented bacteria through Dunnett t-test

| (I) Probiotics | | Mean Difference (I-J) | Std. Error | Sig. | 95% Confidence Interval | |
| --- | --- | --- | --- | --- | --- | --- |
|  |  |  |  |  | Lower Bound | Upper Bound |
| *Lacticaseibacillus rhamnosus* SBBPro6 | Control | -.10140^*^ | .03374 | .040 | -.1987 | -.0041 |
| *Enterococcus faecium* SBBPro7 | Control | -.19540^*^ | .03374 | .000 | -.2927 | -.0981 |
| *Bacillus amyloliquefaciens* SBBPro8 | Control | .02760 | .03374 | .899 | -.0697 | .1249 |
| *Bacillus subtilis* SBBPro9 | Control | -.10640^*^ | .03374 | .030 | -.2037 | -.0091 |
| *Bacillus cereus* SBBPro10 | Control | -.00007 | .03374 | 1.000 | -.0974 | .0972 |

**Supplementary Data Table S9a:** Statistical analysis of cell adhesion assay at incubation time of 30 minutes in current study documented bacteria through One-Sample t-test

| 30min. | | | | |
| --- | --- | --- | --- | --- |
| **One-Sample Statistics** | | | | |
|  | N | Mean | Std. Deviation | Std. Error Mean |
| *Lacticaseibacillus rhamnosus* SBBPro6 | 3 | 40.6667 | .57735 | .33333 |
| *Enterococcus faecium* SBBPro7 | 3 | 40.6667 | .57735 | .33333 |
| *Bacillus amyloliquefaciens* SBBPro8 | 3 | 38.6667 | .57735 | .33333 |
| *Bacillus subtilis* SBBPro9 | 3 | 40.6667 | .57735 | .33333 |
| *Bacillus cereus* SBBPro10 | 3 | 38.0000 | .00000^a^ | 0.00000 |

|  | Test Value = 0 | | | | | |
| --- | --- | --- | --- | --- | --- | --- |
|  | t | df | Sig. (2-tailed) | Mean Difference | 95% Confidence Interval of the Difference | |
|  |  |  |  |  | Lower | Upper |
| *Lacticaseibacillus rhamnosus* SBBPro6 | 122.000 | 2 | .000 | 40.66667 | 39.2324 | 42.1009 |
| *Enterococcus faecium* SBBPro7 | 122.000 | 2 | .000 | 40.66667 | 39.2324 | 42.1009 |
| *Bacillus amyloliquefaciens* SBBPro8 | 116.000 | 2 | .000 | 38.66667 | 37.2324 | 40.1009 |
| *Bacillus subtilis* SBBPro9 | 122.000  122.000 | 2  2 | .000  .000 | 40.66667  40.66667 | 39.2324  39.2324 | 42.1009  42.1009 |

**Supplementary Data Table S9b:** Statistical analysis of cell adhesion assay at incubation time of 60 minutes in current study documented bacteria through One-Sample t-test

| 60min. | | | | |
| --- | --- | --- | --- | --- |
| **One-Sample Statistics** | | | | |
|  | N | Mean | Std. Deviation | Std. Error Mean |
| *Lacticaseibacillus rhamnosus* SBBPro6 | 3 | 58.6667 | .57735 | .33333 |
| *Enterococcus faecium* SBBPro7 | 3 | 57.6667 | 1.15470 | .66667 |
| *Bacillus amyloliquefaciens* SBBPro8 | 3 | 80.6667 | .57735 | .33333 |
| *Bacillus subtilis* SBBPro9 | 3 | 51.6667 | .57735 | .33333 |
| *Bacillus cereus* SBBPro10 | 3 | 46.3333 | 1.15470 | .66667 |

|  | Test Value = 0 | | | | | |
| --- | --- | --- | --- | --- | --- | --- |
|  | t | df | Sig. (2-tailed) | Mean Difference | 95% Confidence Interval of the Difference | |
|  |  |  |  |  | Lower | Upper |
| *Lacticaseibacillus rhamnosus* SBBPro6 | 176.000 | 2 | .000 | 58.66667 | 57.2324 | 60.1009 |
| *Enterococcus faecium* SBBPro7 | 86.500 | 2 | .000 | 57.66667 | 54.7982 | 60.5351 |
| *Bacillus amyloliquefaciens* SBBPro8 | 242.000 | 2 | .000 | 80.66667 | 79.2324 | 82.1009 |
| *Bacillus subtilis* SBBPro9 | 155.000 | 2 | .000 | 51.66667 | 50.2324 | 53.1009 |
| *Bacillus cereus* SBBPro10 | 69.500 | 2 | .000 | 46.33333 | 43.4649 | 49.2018 |

**Supplementary Data Table S9c:** Statistical analysis of cell adhesion assay at incubation time of 90 minutes in current study documented bacteria through One-Sample t-test

| 90min. | | | | |
| --- | --- | --- | --- | --- |
| **One-Sample Statistics** | | | | |
|  | N | Mean | Std. Deviation | Std. Error Mean |
| *Lacticaseibacillus rhamnosus* SBBPro6 | 3 | 127.0000 | 1.00000 | .57735 |
| *Enterococcus faecium* SBBPro7 | 3 | 118.3333 | 1.15470 | .66667 |
| *Bacillus amyloliquefaciens* SBBPro8 | 3 | 124.6667 | .57735 | .33333 |
| *Bacillus subtilis* SBBPro9 | 3 | 128.6667 | .57735 | .33333 |
| *Bacillus cereus* SBBPro10 | 3 | 119.6667 | .57735 | .33333 |

|  | Test Value = 0 | | | | | |
| --- | --- | --- | --- | --- | --- | --- |
|  | t | df | Sig. (2-tailed) | Mean Difference | 95% Confidence Interval of the Difference | |
|  |  |  |  |  | Lower | Upper |
| *Lacticaseibacillus rhamnosus* SBBPro6 | 219.970 | 2 | .000 | 127.00000 | 124.5159 | 129.4841 |
| *Enterococcus faecium* SBBPro7 | 177.500 | 2 | .000 | 118.33333 | 115.4649 | 121.2018 |
| *Bacillus amyloliquefaciens* SBBPro8 | 374.000 | 2 | .000 | 124.66667 | 123.2324 | 126.1009 |
| *Bacillus subtilis* SBBPro9 | 386.000 | 2 | .000 | 128.66667 | 127.2324 | 130.1009 |
| *Bacillus cereus* SBBPro10 | 359.000 | 2 | .000 | 119.66667 | 118.2324 | 121.1009 |

**Supplementary Data Table S10:** Assessment of heat shock tolerance of bacteria isolated in current study through measurement of OD^600^ at log phase after heat shock treatment at 100^o^C

| **Isolates** | **OD^600^ at log phase** | | | **Mean** | **SD** | **p-value** |
| --- | --- | --- | --- | --- | --- | --- |
|  | **I** | **II** | **III** |  |  |  |
| *Lacticaseibacillus rhamnosus* SBBPro6 | 1.433 | 1.396 | 1.388 | 1.40 | ± 0.0240 | 0.05 |
| *Enterococcus faecium* SBBPro7 | 1.305 | 1.301 | 1.303 | 1.303 | ± 0.002 |  |
| *Bacillus amyloliquefaciens* SBBPro8 | 0.969 | 0.953 | 0.950 | 0.957 | ± 0.0102 |  |
| *Bacillus subtilis* SBBPro9 | 1.277 | 1.199 | 1.221 | 1.232 | ± 0.040 |  |
| *Bacillus cereus* SBBPro10 | 1.225 | 1.220 | 1.187 | 1.210 | ± 0.020 |  |

**Supplementary Data Table S11a:** Statistical analysis of heat shock tolerance assay in current study documented bacteria through One-Way Anova

|  | | | | | |
| --- | --- | --- | --- | --- | --- |
|  | Sum of Squares | df | Mean Square | F | Sig. |
| Between Groups | .333 | 5 | .067 | 7.917 | .001 |
| Within Groups | .118 | 14 | .008 |  |  |
| Total | .450 | 19 |  |  |  |

**Supplementary Data Table S11b:** Statistical analysis of heat shock resistance assay in current study documented bacteria through Dunnett t-test

| (I) Probiotics | | Mean Difference (I-J) | Std. Error | Sig. | 95% Confidence Interval | |
| --- | --- | --- | --- | --- | --- | --- |
|  |  |  |  |  | Lower Bound | Upper Bound |
| *Lacticaseibacillus rhamnosus* SBBPro6 | Control | .16907 | .06694 | .097 | -.0239 | .3620 |
| *Enterococcus faecium* SBBPro7 | Control | .06640 | .06694 | .811 | -.1266 | .2594 |
| *Bacillus amyloliquefaciens* SBBPro8 | Control | -.27927^*^ | .06694 | .004 | -.4722 | -.0863 |
| *Bacillus subtilis* SBBPro9 | Control | -.00427 | .06694 | 1.000 | -.1972 | .1887 |
| *Bacillus cereus* SBBPro10 | Control | -.02593 | .06694 | .995 | -.2189 | .1670 |

**Supplementary Data Table S12:** Antibiotic sensitivity profiling of current study documented bacteria against different antibiotics through measurement of zones of inhibition

| **Isolates** | **Azithromycin** | | | | | **Erythromycin** | | | | | **Amoxil** | | | | |
| --- | --- | --- | --- | --- | --- | --- | --- | --- | --- | --- | --- | --- | --- | --- | --- |
|  | 10ul | 20ul | 30ul | mean | SD | 10ul | 20ul | 30ul | mean | SD | 10ul | 20ul | 30ul | mean | SD |
| *Lacticaseibacillus rhamnosus* SBBPro6 | 10 | 13 | 11 | 11.33 | ± 0.002 | 11 | 9 | 13 | 11 | ± 0.001 | 10 | 12 | 14 | 12 | ± 0.001 |
| *Enterococcus faecium* SBBPro7 | 8.5 | 13.5 | 11 | 11 | ± 0.001 | 5.5 | 6 | 6.5 | 6 | ± 0.001 | 12.5 | 10 | 12 | 11.5 | ± 0.001 |
| *Bacillus amyloliquefaciens* SBBPro8 | 7.5 | 8.5 | 6.5 | 7.5 | ± 0.001 | 4.5 | 4.5 | 11 | 6.66 | ± 0.002 | 10 | 11.5 | 16 | 12.5 | ± 0.002 |
| *Bacillus subtilis* SBBPro9 | 5 | 12 | 16 | 11 | ± 0.002 | 9.5 | 10 | 12.5 | 10.6 | ± 0.001 | 9.5 | 13.5 | 12 | 11.6 | ± 0.001 |
| *Bacillus cereus* SBBPro10 | 3.5 | 5 | 18 | 8.83 | ± 0.001 | 6.5 | 9.5 | 11.5 | 9.16 | ± 0.002 | 10 | 12 | 13 | 11.6 | ± 0.001 |

| **Isolates** | **Ciprofloxacin** | | | | | **Velosef** | | | | |
| --- | --- | --- | --- | --- | --- | --- | --- | --- | --- | --- |
|  | 10ul | 20ul | 30ul | mean | SD | 10ul | 20ul | 30ul | mean | SD |
| *Lacticaseibacillus rhamnosus* SBBPro6 | 14.5 | 16 | 15 | 15.1 | ± 0.002 | 31.5 | 30.5 | 32.5 | 31.5 | ± 0.0240 |
| *Enterococcus faecium* SBBPro7 | 14 | 13.5 | 12.5 | 13.3 | ± 0.0210 | 30.5 | 31.5 | 33 | 31.6 | ± 0.0102 |
| *Bacillus amyloliquefaciens* SBBPro8 | 16.5 | 17.5 | 16.5 | 16.8 | ± 0.001 | 33.5 | 33.5 | 33.5 | 33.5 | ± 0.0240 |
| *Bacillus subtilis* SBBPro9 | 14.5 | 13.5 | 10.5 | 12.8 | ± 0.002 | 33.5 | 35 | 37.5 | 35.3 | ± 0.0140 |
| *Bacillus cereus* SBBPro10 | 15 | 15 | 15 | 15 | ± 0.001 | 32 | 34 | 36.5 | 34.1 | ± 0.0220 |

**Supplementary Data Table S13a:** Statistical analysis of antibiotic resistance assay against azithromycin in current study documented bacteria through One-Sample t-test

| **One-Sample Statistics** | | | | |  |  |
| --- | --- | --- | --- | --- | --- | --- |
| Concentration (µl) | N | Mean | Std. Deviation | Std. Error Mean |  |  |
| 10 | 5 | 6.9000 | 2.63154 | 1.17686 |  |  |
| 20 | 5 | 10.4000 | 3.59514 | 1.60779 |  |  |
| 30 | 5 | 12.5000 | 4.55522 | 2.03715 |  |  |
|  |  |  |  |  |  |  |
|  |  |  |  |  |  |  |
| **One-Sample Test** | | | | | | |
| Concentration (µl) | Test Value = 0 | | | | | |
|  | t | df | Sig. (2-tailed) | Mean Difference | 95% Confidence Interval of the Difference | |
|  |  |  |  |  | Lower | Upper |
| 10 | 5.863 | 4 | .004 | 6.90000 | 3.6325 | 10.1675 |
| 20 | 6.468 | 4 | .003 | 10.40000 | 5.9360 | 14.8640 |
| 30 | 6.136 | 4 | .004 | 12.50000 | 6.8440 | 18.1560 |
|  |  |  |  |  |  |  |

**Supplementary Data Table S13b:** Statistical analysis of antibiotic resistance assay against erythromycin in current study documented bacteria through One-Sample t-test

|  | **One-Sample Statistics** | | | | |  |  |
| --- | --- | --- | --- | --- | --- | --- | --- |
|  | Concentration (µl) | N | Mean | Std. Deviation | Std. Error Mean |  |  |
|  | 10 | 5 | 7.4000 | 2.74773 | 1.22882 |  |  |
|  | 20 | 5 | 7.8000 | 2.41350 | 1.07935 |  |  |
|  | 30 | 5 | 10.9000 | 2.58360 | 1.15542 |  |  |
|  |  |  |  |  |  |  |  |
|  |  |  |  |  |  |  |  |
|  | **One-Sample Test** | | | | | | |
|  | Concentration (µl) | Test Value = 0 | | | | | |
|  |  | t | df | Sig. (2-tailed) | Mean Difference | 95% Confidence Interval of the Difference | |
|  |  |  |  |  |  | Lower | Upper |
|  | 10 | 6.022 | 4 | .004 | 7.40000 | 3.9882 | 10.8118 |
|  | 20 | 7.227 | 4 | .002 | 7.80000 | 4.8032 | 10.7968 |
|  | 30 | 9.434 | 4 | .001 | 10.90000 | 7.6920 | 14.1080 |

**Supplementary Data Table S13c:** Statistical analysis of antibiotic resistance assay against amoxil in current study documented bacteria through One-Sample t-test

| **One-Sample Statistics** | | | | |  |  |
| --- | --- | --- | --- | --- | --- | --- |
| Concentration (µl) | N | Mean | Std. Deviation | Std. Error Mean |  |  |
| 10 | 5 | 10.4000 | 1.19373 | .53385 |  |  |
| 20 | 5 | 11.8000 | 1.25499 | .56125 |  |  |
| 30 | 5 | 13.4000 | 1.67332 | .74833 |  |  |
|  |  |  |  |  |  |  |
|  |  |  |  |  |  |  |
| **One-Sample Test** | | | | | | |
| Concentration (µl) | Test Value = 0 | | | | | |
|  | t | df | Sig. (2-tailed) | Mean Difference | 95% Confidence Interval of the Difference | |
|  |  |  |  |  | Lower | Upper |
| 10 | 19.481 | 4 | .000 | 10.40000 | 8.9178 | 11.8822 |
| 20 | 21.025 | 4 | .000 | 11.80000 | 10.2417 | 13.3583 |
| 30 | 17.907 | 4 | .000 | 13.40000 | 11.3223 | 15.4777 |

**Supplementary Data Table S13d:** Statistical analysis of antibiotic resistance assay against ciprofloxacin in current study documented bacteria through One-Sample t-test

| **One-Sample Statistics** | | | | |  |  |
| --- | --- | --- | --- | --- | --- | --- |
| Concentration (µl) | N | Mean | Std. Deviation | Std. Error Mean |  |  |
| 10 | 5 | 14.9000 | .96177 | .43012 |  |  |
| 20 | 5 | 15.1000 | 1.71026 | .76485 |  |  |
| 30 | 5 | 13.9000 | 2.38223 | 1.06536 |  |  |
|  |  |  |  |  |  |  |
|  |  |  |  |  |  |  |
|  |  |  |  |  |  |  |
| **One-Sample Test** | | | | | | |
| Concentration (µl) | Test Value = 0 | | | | | |
|  | t | df | Sig. (2-tailed) | Mean Difference | 95% Confidence Interval of the Difference | |
|  |  |  |  |  | Lower | Upper |
| 10 | 34.642 | 4 | .000 | 14.90000 | 13.7058 | 16.0942 |
| 20 | 19.742 | 4 | .000 | 15.10000 | 12.9764 | 17.2236 |
| 30 | 13.047 | 4 | .000 | 13.90000 | 10.9421 | 16.8579 |

**Supplementary Data Table S13e:** Statistical analysis of antibiotic resistance assay against velosef in current study documented bacteria through One-Sample t-test

| **One-Sample Statistics** | | | | |  |  |  |
| --- | --- | --- | --- | --- | --- | --- | --- |
| Concentration (µl) | N | Mean | Std. Deviation | Std. Error Mean |  |  |  |
| 10 | 5 | 32.2000 | 1.30384 | .58310 |  |  |  |
| 20 | 5 | 32.9000 | 1.85068 | .82765 |  |  |  |
| 30 | 5 | 34.6000 | 2.24722 | 1.00499 |  |  |  |
|  |  |  |  |  |  |  |  |
|  |  |  |  |  |  |  |  |
|  |  |  |  |  |  |  |  |
| **One-Sample Test** | | | | | | |  |
| Concentration (µl) | Test Value = 0 | | | | | |  |
|  | t | df | Sig. (2-tailed) | Mean Difference | 95% Confidence Interval of the Difference | |  |
|  |  |  |  |  | Lower | Upper |  |
| 10 | 55.223 | 4 | .000 | 32.20000 | 30.5811 | 33.8189 |  |
| 20 | 39.751 | 4 | .000 | 32.90000 | 30.6021 | 35.1979 |  |
| 30 | 34.428 | 4 | .000 | 34.60000 | 31.8097 | 37.3903 |  |
